# Cysteine-Rich Angiogenic Inducer 61: Pro-Survival Function and Role as a Biomarker for Disseminating Breast Cancer Cells

**DOI:** 10.3390/cancers13030563

**Published:** 2021-02-02

**Authors:** Kai Bartkowiak, Isabel Heidrich, Marcel Kwiatkowski, Tobias M. Gorges, Antje Andreas, Maria Geffken, Karl Verpoort, Volkmar Müller, Hartmut Schlüter, Klaus Pantel

**Affiliations:** 1 Department of Tumor Biology, University Medical Centre Hamburg-Eppendorf, Martinistraße 52, 20246 Hamburg, Germany; kbartkowiak@uke.de (K.B.); info-tumorbiologie@uke.de (I.H.); t.gorges@uke.de (T.M.G.); a.andreas@uke.de (A.A.); 2 Laboratory for Metabolic Signaling, Institute of Biochemistry, University of Innsbruck, Innrain 80-82, 6020 Innsbruck, Austria; Marcel.Kwiatkowski@uibk.ac.at; 3 Department of Transfusion Medicine, University Medical Centre Hamburg-Eppendorf, Martinistraße 52, 20246 Hamburg, Germany; Maria.Geffken@uke.de; 4 Practice for Haematology and Oncology, Hohe Weide 17b, 20295 Hamburg, Germany; k.verpoort@t-online.de; 5 Department of Gynecology, University Medical Center Hamburg-Eppendorf, Martinistraße 52, 20246 Hamburg, Germany; v.mueller@uke.de; 6 Institute of Clinical Chemistry and Laboratory Medicine, University Medical Centre Hamburg-Eppendorf, Martinistraße 52, 20246 Hamburg, Germany; hschluet@uke.de

**Keywords:** breast cancer, dissemination, circulating tumor cells, hypoxia

## Abstract

**Simple Summary:**

Metastasis is the leading cause of death in breast cancer, and it can be predicted by the detection of circulating tumor cells in the blood and disseminated tumor cells in the bone marrow, which are usually detected by epithelial marker proteins. However, tumor cells with mesenchymal attributes down-regulate the expression of epithelial marker proteins, and are therefore difficult to detect. Here, we found that the protein-cysteine–rich angiogenetic inducer 61 (Cyr61) is strongly expressed in tumor cells with mesenchymal attributes. Cyr61 expression was undetectable in normal blood cells, suggesting that Cyr61 might represent a tumor-associated protein. Functional experiments showed that the loss of Cyr61 reduces the viability of breast tumor cells. Thus, Cyr61 might represent an interesting anti-metastatic target that needs to be explored in future studies.

**Abstract:**

(1) Background: the early detection of cancer cells in the blood or bone marrow of breast cancer patients improves the understanding of metastasis. Disseminating tumor cells in the bone marrow with a pronounced manifestation of mesenchymal markers (mDTC) are difficult to detect by epithelial markers, but they are relevant in the initiation of metastasis. (2) Methods: the breast cancer mDTC cell line BC-M1 was analyzed by mass spectrometry, which revealed high levels of the protein-cysteine–rich angiogenic inducer 61 (Cyr61). The function of Cyr61 was investigated using shRNA and hypoxia. Peripheral blood samples from 35 breast cancer patients were investigated for CTCs defined as cytokeratin-positive/CD45-negative cells. (3) Results: the Cyr61 levels are elevated in mDTC lines from breast, lung, and prostate cancer patients. The loss of Cyr61 resulted in the diminished expression of hypoxia-inducible factor 1-alpha, and increased apoptosis. Cyr61 was present in 47 (43%) of the 109 detected circulating tumor cells (CTCs), while the blood and bone marrow cells from healthy controls were Cyr61-negative. (4) Conclusions: Cyr61 is expressed in mDTC lines, supports the viability of cancer cells, and classifies a new subset of cytokeratin-positive CTCs, which deserves further investigation.

## 1. Introduction

The ‘liquid biopsy’ analysis of the peripheral blood of cancer patients has made remarkable progress [1]. The subjects of liquid biopsy are circulating tumor cells (CTCs) or molecules like nucleic acids or proteins that are released by tumor cells into the blood. When such tumor-derived biomaterial is detected in the blood, it can be used for the early detection of cancer, the prediction of metastatic relapse, or therapy monitoring [2].

In breast cancer, the detection of CTCs in the blood, and their relatives that have already reached secondary sites—the disseminated tumor cells in the bone marrow (DTCs)—are useful markers for the prediction of distant metastasis [3]. DTCs survive chemotherapy and predict poor clinical outcomes of the patients [4,5,6,7]. However, there is still a substantial number of patients who relapse despite negative blood (CTCs) or bone marrow (DTCs) findings at their primary diagnosis [5,8,9], reflecting the heterogeneity of the disseminating tumor cells [3]. However, most studies have employed antibodies against epithelial cell markers in order to detect CTCs or DTCs [10]. Besides breast carcinoma with an epithelial phenotype, there is also a fluent transition of phenotypes with varying degrees of mesenchymal attributes [11,12,13]. Hence, due to a reduced moulding of epithelial characteristics, DTCs and CTCs with the pronounced manifestation of mesenchymal attributes and a weak expression of epithelial markers (mDTC and mCTC) are difficult to detect by epithelial cell markers. Recent experiments have confirmed the presence of such CTCs in breast [14] and lung cancer patients [15]. Thus, there is an urgent need for new markers that support the detection of mDTCs and mCTCs in routine analysis.

One factor that can promote the acquisition of mesenchymal attributes and the dissemination of tumor cells is hypoxia [16,17]. Hypoxia can be present in the primary tumor and in secondary metastatic sites like the bone marrow, where concentrations of only 1% O_2_ (hypoxia) are detected [18,19]. We previously detected microenvironmental stress-tolerant mDTCs in the bone marrow that were strongly positive for proteins of hypoxic stress-response programs [20]. Hence, the proteins of stress response programs could be applicable as markers for the detection of mCTCs and mDTCs that are difficult to identify by the established markers used in previous studies.

The 42 kDa protein cysteine-rich angiogenic inducer 61 (Cyr61) is a versatile responder for microenvironmental stress, and can be secreted to the extracellular space. Cyr61 has been implicated in migration, cellular differentiation, and the induction of angiogenesis [21]. In breast cancer, about thirty percent of invasive tumors show elevated Cyr61 expression compared to normal tissue, and higher levels of Cyr61 are associated with the formation of metastasis [22,23]. The promotion of metastasis by Cyr61 is mediated by enabling tumor cell extravasation and protecting tumor cells from anoikis during the dissemination process [24]. However, the detailed analysis of the pathophysiologic role of Cyr61 is complicated by its complex and dynamic regulation, leading to diverse and sometimes opposing cellular responses depending on the individual cell context [25].

Three major factors were discovered that influence the expression of Cyr61. First, the stimulation of the epidermal growth factor receptor (EGFR) can induce an increase in cytoplasmic Cyr61 levels [26], while the effect on secreted Cyr61 was not investigated. Another function of Cyr61 which is relevant in metastasis is that Cyr61 is a hypoxia-inducible angiogenesis factor which might interact with hypoxia-inducible factor-1-alpha (HIF-1α) as a master regulator of the metabolic adaptation to hypoxia. This effect might support the idea of a hypoxia-induced selection process during tumor progression towards tumor cells with elevated Cyr61 expression [27]. Secreted Cyr61 can act as a ligand for different integrin species, of which the integrin αv/integrin β3 heterodimer appears to be the favoured receptor for Cyr61 [22].

We have previously observed that breast cancer cells with low keratin expression due to an epithelial–mesenchymal transition show elevated levels of the immune checkpoint regulator programmed death-ligand 1 (PD-L1), and that PD-L1 is induced by hypoxia in such cells [28]. Therapies that are directed against the control of the immune checkpoints have become interesting novel approaches, and PD-L1 is currently one of the most prominent therapeutic targets [29]. PD-L1 has been detected in CTCs using keratins as CTC-detection proteins [30]. However, it might be possible that additional breast cancer cells that only weakly express keratins display high levels of Cyr61 and PD-L1, and are therefore overlooked by standard CTC assays. In particular, the remarkably-quick response of PD-L1 to changing oxygen concentrations [28] attracted our interest here to study a potential link between Cyr61 and PD-L1. To our best knowledge, no reports have been published on Cyr61 expression in the CTCs or DTCs of cancer patients.

The principal goal of this study was to determine whether Cyr61 may constitute a robust marker for CTC/DTC, and we therefore aimed to examine the fluctuations of Cyr61 levels in various tumor cell lines and DTC cell lines cultivated in different conditions that mimic various microenvironmental stresses encountered by CTCs and DTCs. First, we used a mass-spectrometry–based approach that revealed the protein Cyr61 in mDTC cell lines. We then investigated which common biological parameters that are relevant to breast cancer progression (e.g., ErbB-2, microenvironmental stress) affect the cytoplasmic levels of Cyr61 in tumor cells. These results provide important information on the expected range of Cyr61 levels in CTC or DTC in clinical samples from the breast cancer patients analysed in this investigation.

## 2. Results

### 2.1. Identification of Cyr61 in DTC Cell Lines

The DTC cell lines with mesenchymal attributes from the bone marrow of cancer patients (BC-M1: breast cancer; LC-M1: lung cancer; PC-E1: prostate cancer) served as useful models to study the biology of DTCs [20,31,32,33]. We compared the protein levels of BC-M1 with the basal-like breast cancer cell line MDA-MB-468 in a mass-spectrometry–based approach using SILAC (stable isotope labelling with amino acids in cell culture). For the analysis of the proteins that might be relevant in the dissemination process, we selected the triple-negative basal-like breast cancer cell line MDA-MB-468 as the reference cell line for the comparison with BC-M1. Since we observed that MDA-MB-468 can induce vimentin under hypoxia [28], we assumed that MDA-MB-468 might contain a subpopulation of tumor cells that may acquire mesenchymal attributes under cell stress.

**Figure 1:**
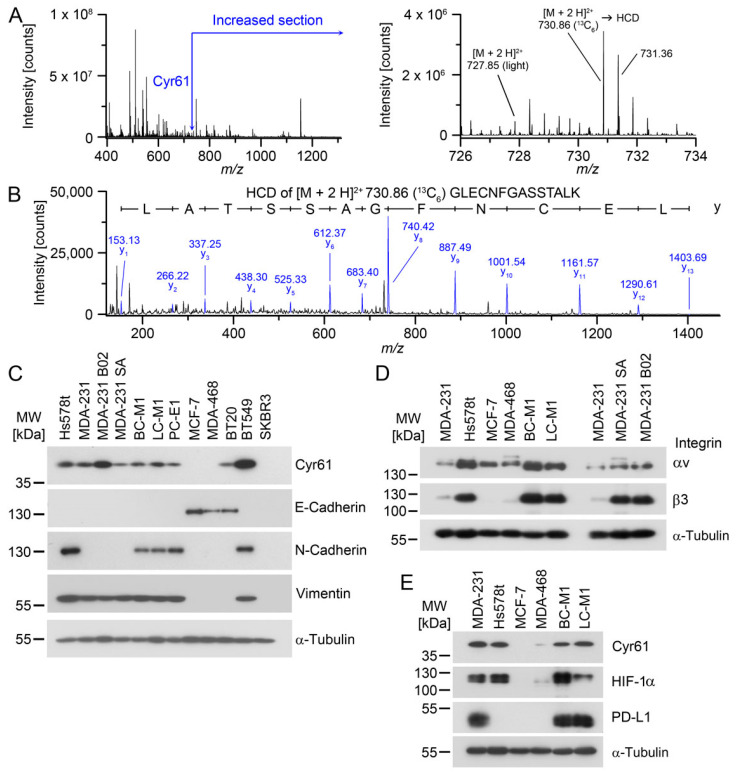
Detection of Cyr61 in tumor cell lines with mesenchymal attributes. (**A**) Detection of Cyr61 by SILAC LC-MS/MS. Left: MS_1_ (survey scan) mass spectrum containing the peptides around m/z 731 Da. Right: enlarged section of the Cyr61 peptides containing the light masses from MDA-MB-468 and the ^13^C_6_-labelled peptides of BC-M1. (**B**) Positive ion mode HCD (higher-energy collisional dissociation) spectrum of m/z 730.86 [M + 3 H]^3+^ Da. The relevant fragments of the y-ion series are assigned with their masses. (**C**) Confirmation of the differential expression of Cyr61 and comparison with the epithelial grade in breast cancer cell lines or DTC cell lines (BC-M1, LC-M1, PC-E1) by Western blot analysis. (**D**) Detection of the integrin species in the breast cancer cell lines by Western blot analysis. (**E**) Comparison of the protein levels of Cyr61, HIF-1α and PD-L1 in the breast cancer cell lines, as detected by Western blot analysis. (**A**,**B**) n_biol_ = 4; (**C**–**E**): n_biol_ = 3.

For the six Cyr61 peptides that could be reliably quantified, the levels were 12.51 ± 2.24-fold higher in BC-M1 than in MDA-MB-468 (Figure 1A,B; Table S1). We confirmed the levels of Cyr61 by Western blot analysis, and observed that the cell lines with mesenchymal attributes or an intermediate epithelial/mesenchymal phenotype were positive for Cyr61 (Figure 1C, Figure S1). Since Cyr61 can be secreted into the extracellular space, and acts there as a ligand, we investigated the expression levels of the preferred receptors of Cyr61 of the integrin family. The proteins that constitute the integrin αv/integrin β3 heterodimer are present at high levels in the DTC cell lines BC-M1 and LC-M1 (Figure 1D). Due to the remarkably-high levels of these integrins in the analysed cell lines, we investigated whether the dissemination of tumor cells to the bone marrow might affect the levels of integrin αv and integrin β3. For this purpose, we analysed the bone metastasis cell lines MDA-MB-231 B02 (B02) and MDA-MB-231 SA (SA) derived from the parental breast cancer cell line MDA-MB-231 (Figure 1D). The integrin αv levels were slightly increased in B02 and SA compared with MDA-MB-231. In contrast, we observed strongly-elevated levels of integrin β3 in B02 and SA compared with MDA-MB-231.

Unlike the other organs, bone marrow exhibits hypoxic areas adjacent to the bone surface even under physiologic conditions, which might be suitable dissemination sites for hypoxia-adapted tumor cells. Therefore, we compared the protein levels of Cyr61 with the master regulator of metabolic adaptation to hypoxia HIF-1α (Figure 1E). We observed that cell lines that were positive for Cyr61 were also positive for HIF-1α in breast cancer (MDA-MB-231, Hs578t, MDA-MB-468) and DTC cell lines (BC-M1, LC-M1), suggesting a potential regulative interaction of HIF-1α with Cyr61.

Since we previously noticed that PD-L1 is induced under hypoxia [28], we further explored whether Cyr61/HIF-1α double positive cells also express PD-L1. PD-L1 can block the activation of T-cells as an immune checkpoint molecule [34]. PD-L1 has also become a key target for immunotherapies in solid tumors, and is expressed in CTCs in breast cancer [30]. We found that three cell lines that were double positive for Cyr61/HIF-1α were also positive for PD-L1 (Figure 1E).

### 2.2. Cyr61 Responses to Microenvironmental Stress

In order to obtain additional information on whether Cyr61 can be applied as a detection marker for CTC and DTC, we investigated the typical microenvironmental conditions that are common under ischemia in breast tumors, and determined the changes of the Cyr61 levels in response to these conditions.

**Figure 2:**
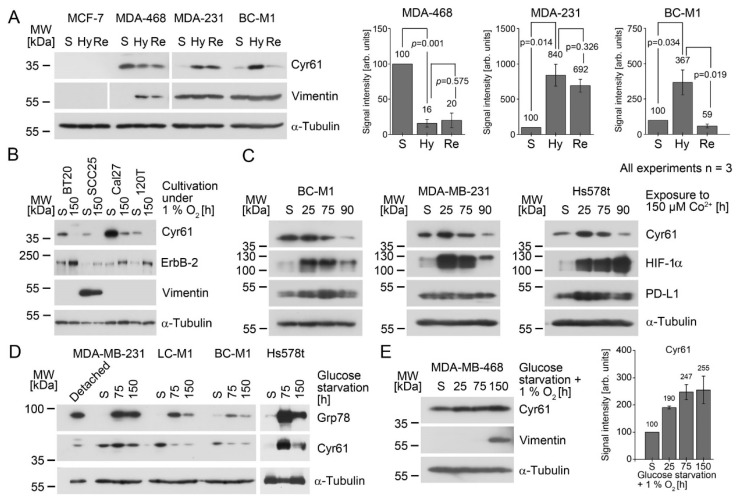
Response of the Cyr61 levels to hostile microenvironmental conditions; analyses by Western blot analysis. The starting conditions for all of the experiments were the standard cell culture conditions (S). (**A**) Response of Cyr61 levels to chronic hypoxia (14 days 1% O_2_; condition Hy) and reoxygenation (Re: Hy followed by 10% O_2_ for 4 h). The statistical analyses of the changes in the protein levels are shown in the bar diagrams (right), and the obtained *p*-values are presented as numbers. The *p*-values were calculated using Student’s *t*-test (n_biol_ = 3). The corresponding scatterplot is shown in Figure S2. (**B**) Downregulation of Cyr61 under hypoxia (1% O_2_) and the induction of ErbB-2 under hypoxia. (**C**) Effect of the stabilization of HIF-1α to Cyr61 and PD-L1 levels after the treatment of the cells with cobalt chloride. (**D**) Effect of glucose starvation on the Cyr61 levels. As a large amount of MDA-MB-231 cells detached from the cell culture flask after 50 h of glucose withdrawal, the detached cells were analysed separately. The 78 kDa glucose-regulated protein (Grp78) was analysed as a positive control for a cellular response after glucose withdrawal. (**E**) Induction of Cyr61 under hypoxia and glucose starvation. The diagrams display the quantitative analyses of the Cyr61 levels of the Western blot images (n_biol_ = 3). The numbers above the error bars (standard deviation) represent the average values. The corresponding scatterplot is shown in Figure S3.

The observation that Cyr61 positive cell lines were also positive for HIF-1α suggested that Cyr61 might be induced under hypoxic conditions. In order to explore this further, we subjected a set of breast cancer cell lines to hypoxia (1% O_2_). Tumor cells may reside in hypoxic areas of the primary tumor or at metastatic sites before they enter the well-oxygenated blood stream, which shows an oxygen concentration of ±10% O_2_ [35]. After 4 h, approximately two-thirds of the CTC have departed from the blood circuit [36]. In order to model this situation, we subjected breast cancer cell lines to chronic hypoxia for 14 days, followed by a reoxygenation pulse of 10% O_2_ for 4 h, and analysed the Cyr61 levels (Figure 2A). Under hypoxia, Cyr61 was induced in MDA-MB-231 and BC-M1 cells, which would resemble the condition in a hypoxic tissue microenvironment. After re-oxygenation, which would mimic the release of CTCs into the circulation, Cyr61 was downregulated in BC-M1 cells, whereas in MDA-MB-231, no significant change in the Cyr61 levels was detected after the reoxygenation. Thus, hypoxic BC-M1 cells would appear strongly Cyr61 positive cells at the start of the dissemination, and may remain detectable when they depart from the circulation, yet with lower signal intensity. Unlike MDA-MB-231 and BC-M1, in MDA-MB-468 a significant downregulation of Cyr61 was observed under hypoxia compared with the standard cell culture condition (Figure 2A). Since we previously observed that MDA-MB-468 induces ErbB-2 under hypoxia [28], whereas MDA-MB-231 and BC-M1 did not, we tried to identify additional cell lines that show an induction of ErbB-2 with the concomitant downregulation of Cyr61. We screened cell lines for ErbB-2 induction under hypoxia. All four cell lines identified showed reduced Cyr61 levels under hypoxia with the concomitant induction of ErbB-2 (Figure 2B), suggesting that ErbB-2 might be involved in the downregulation of Cyr61 under hypoxia.

Since hypoxia induces a variety of different cellular adaptation strategies, it is difficult to assign the response of Cyr61 to hypoxia to a specific program. In order to obtain additional insights, we treated the cells with cobalt chloride in the presence of standard cell culture conditions. Cobalt (Co^2+^) inhibits the interaction of HIF-1α with the von Hippel–Lindau protein, leading to an accumulation of HIF-1α [37]. These experiments revealed an accumulation of HIF-1α, with the subsequent downregulation of Cyr61 (Figure 2C). Furthermore, we investigated the cellular response of PD-L1 after the treatment of the cells with cobalt chloride. We observed a transient induction of PD-L1 in BC-M1 and Hs578t after the treatment of the cells with cobalt chloride for 25 h, but after 90 h of this treatment, the PD-L1 levels were again comparable to the initial values. For MDA-MB-231 cells, we could not detect considerable changes in the PD-L1 levels upon treatment with cobalt chloride. This suggests that PD-L1 might be regulated by HIF-1α, but less tightly than Cyr61.

Inefficient blood supply in tumors is frequently accompanied by a lack of nutrients. Therefore, we analysed the cellular response to glucose starvation. The Cyr61 levels in Hs578t were strongly induced under glucose starvation, whereas a downregulation of Cyr61 was detected for BC-M1 and LC-M1, and no appreciable changes in the Cyr61 levels were observed for MDA-MB-231 (Figure 2D). We next selected MDA-MB-468—in which a Cyr61 downregulation was observed under hypoxia (Figure 2A)—and subjected the cells to hypoxia and glucose starvation together, which also lead to the induction of Cyr61 in these cells (Figure 2E).

These experiments suggest that Cyr61 levels may be altered under microenvironmental stress. However, the reaction might depend on the presence of ErbB-2 or HIF-1α, or the particular cell phenotype or the inducing microenvironmental stress factor. Generally, the Cyr61 levels under hypoxic conditions (including elevated levels of HIF-1α) might still be sufficient for CTC/DTC detection. However, the combination of hypoxia and ErbB-2 induction leads to the reduction of the Cyr61 levels, which might hinder the detection of those CTC/DTC. Furthermore, glucose starvation—as found in the ischemic regions of the tumor—maintains Cyr61 levels that are still suitable for the application of Cyr61 as a marker protein, with the exception of phenotypes like BC-M1 and LC-M1.

### 2.3. Cyr61 Knockdown Diminishes the Viability of Tumor Cells

The previous experiments showed the dynamic behavior of Cyr61 in response to different stimuli and microenvironmental conditions, but it was difficult to assign Cyr61 levels to a specific phenotype associated with dissemination. Such an allocation would provide valuable information about the attributes of the Cyr61-positive CTC/DTC phenotypes detected in cancer patients.

**Figure 3:**
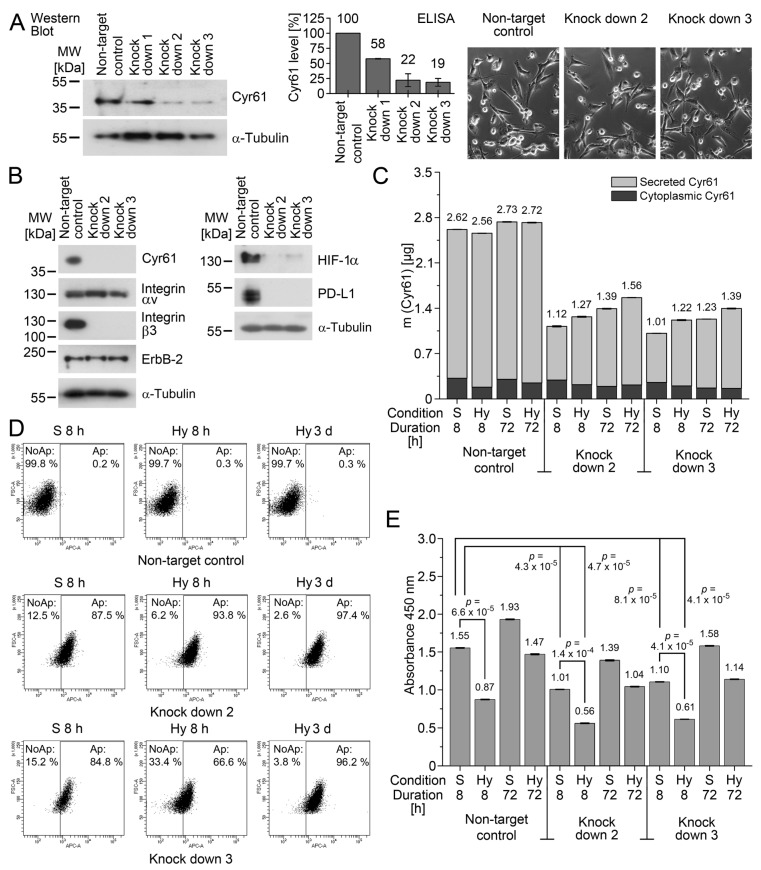
Cyr61 knockdown in MDA-MB-231 cultured under standard conditions (S) and under hypoxia (1% O_2_; Hy). (**A**) Establishment of Cyr61 knockdowns in MDA-MB-231 by shRNA. The cytoplasmic Cyr61 knockdown was detected by Western blot (left), quantified by ELISA (diagram), and analysed for morphologic changes (photomicrographs, right). The corresponding scatterplot is shown in Figure S4. (**B**) Cyr61 knockdown in MDA-MB-231 by shRNA, analysed by Western blot analysis. (**C**) Analysis of cytoplasmic and secreted Cyr61 by ELISA (n_biol_ = 3). The numbers refer to the sum values of the cytoplasmic and secreted Cyr61. The corresponding scatterplot is shown in Figure S5. (**D**) The apoptosis rate in the cells detected by APC (allophycocyanin)-labelled Annexin V, and the relative cell size (FSC-A). The percentages of non-apoptotic (NoAp) and apoptotic (Ap) cells are denoted as numbers for each experiment. (**E**) Bromodeoxyuridine (BrdU) assay for the analysis of the cell proliferation. The *p*-values (given as numbers) were calculated using Student’s *t*-test (n_biol_ = 3). The corresponding scatterplot is shown in Figure S6.

To study this, we selected MDA-MB-231 as a model, as this cell line frequently forms metastases in the bones of mice. Cyr61 was downregulated by shRNA in MDA-MB-231 (Figure 3A), and the extent of the knockdown was quantified by an enzyme-linked immunosorbent assay (ELISA) for Cyr61. The knockdowns #2 and #3 were selected for further analysis, and these cells showed no detectable morphologic changes compared with the non-target control cells (Figure 3A). Similarly to previous findings [38], we detected reduced levels of the integrin α_v_β_3_ heterodimer in MDA-MB-231 (Figure 3B). Moreover, we found that the knockdown of Cyr61 resulted in the downregulation of HIF-1α, which supports the idea that Cyr61 might be involved in the regulation of HIF-1α. We also noticed that the knockdown of Cyr61 leads to reduced levels of PD-L1, which supports the idea that Cyr61-positive CTC and DTC are also positive for PD-L1. Future investigations need to be performed in order to clarify whether there is a mechanistic link between Cyr61 and PDL1.

As we were particularly interested in adaptation strategies during the early stage of the tumor cells’ settlement in the bone marrow, we subjected the cells to hypoxia for 8 h. In order to investigate whether the cells reach a stable state later on, we also investigated the cells after 72 h of hypoxia. Next, the total amount of Cyr61 comprising cytoplasmic and secreted Cyr61 under the standard culture conditions and hypoxia (1% O_2_) was determined by ELISA (Figure 3C). Increased early apoptosis was observed in the knockdown cells under the standard culture conditions and hypoxia compared with the non-target control (Figure 3D). Under the standard culture conditions, the Cyr61 knockdown resulted in reduced cell proliferation (Figure 3E). As Annexin V staining detects early stages of apoptosis, it might be possible that apoptosis-induced biochemical changes in the cells might not be visible by the cell shape (Figure 3A). Similarly, the bromodeoxyuridine assay sensitively detects the reduced novel synthesis of DNA, in particular under acute hypoxia in our experiments (8 h); however, as the transiently low-proliferating cells are able to recover, this effect was not clearly seen by the visual inspection of the cells. Acute hypoxia (8 h) leads to reduced proliferation rates both in the knockdown and the control cells, but proliferation rates increased again after 72 h of hypoxia.

These findings suggest that the knockdown of Cyr61 induced elevated apoptosis and low proliferation, and that both Cyr61 knockdown and control cells respond similarly to hypoxia (namely a reduction in proliferation and slight increases in apoptosis).

### 2.4. Cyr61 Secretion in MDA-MB-231 and BC-M1

CTC and DTC detection by Cyr61 relies on the presence of cytoplasmic Cyr61. However, Cyr61 can be secreted, which may reduce the amount of cytoplasmic Cyr61 available for immunocytochemical staining. Thus, we modeled a considerable secretion of Cyr61 into the extracellular space through epidermal growth factor (EGF) stimulation, and measured in parallel the cytoplasmic Cyr61 concentrations.

**Figure 4:**
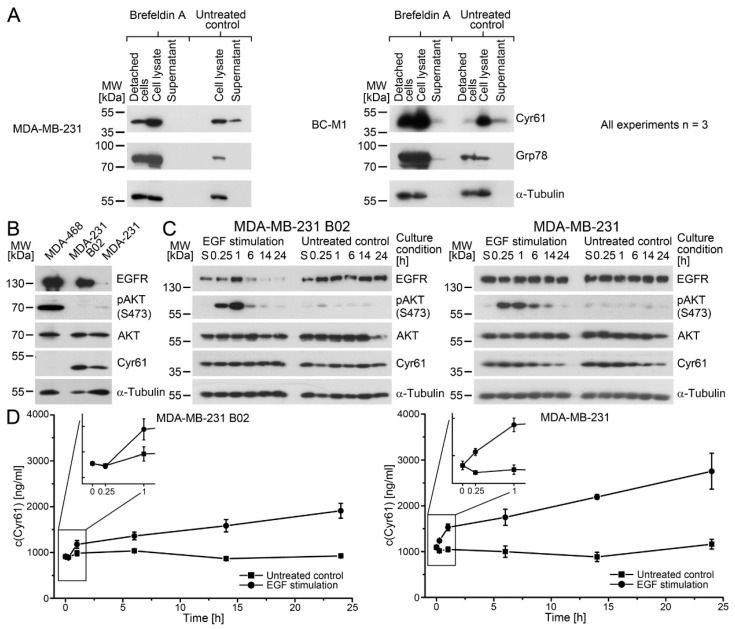
Cyr61 secretion in breast cancer cells. (**A**) Detection of Cyr61 secretion in MDA-MB-231 (MDA-231) and BC-M1 by Western blot analysis. Cells were treated with Brefeldin A to block the protein secretion. The supernatant of the cell culture medium contains the secreted protein fraction, whereas the pellet contains the detached cells. As the total protein amount for two of the three biological replicates of the MDA-231 detached cells (untreated) was below 50 µg, a Western blot lacking this sample is shown. The 78 kDa glucose-regulated protein (Grp78) was analysed as the positive control for a cellular response to Brefeldin A treatment. (**B**) A comparison of the protein levels in the parental cell line MDA-231 and its bone metastatic subline MDA-231 B02 in the non-stimulated state by Western blot analysis. (**C**) The activation of the cells (detected as pAKT S473) upon EGF stimulation, and the analysis of the cytoplasmic Cyr61 levels by Western blot analysis. (**D**) The secreted Cyr61 levels in the cell culture supernatants after EGF stimulation. For a statistical analysis of the values, see Tables S2 and S3. The corresponding scatterplots are shown in Figure S7.

In a first step, we analyzed the cell culture medium of MDA-MB-231 and BC-M1 for the presence of secreted Cyr61 (Figure 4A). As it cannot be excluded that lysed cells may release Cyr61, the cells were treated with Brefeldin A (BFA) in parallel. BFA inhibits the protein transport from the endoplasmic reticulum to the Golgi apparatus, which leads to an accumulation of secreted proteins in the endoplasmic reticulum. In order to clear the cell culture medium from the detached cells, the culture medium was centrifuged. The soluble fraction was designated as the supernatant, and the insoluble debris were designated as detached cells. Elevated Cyr61 signals in the untreated supernatant control compared with the corresponding BFA treated fraction were detected in MDA-MB-231 and BC-M1 by Western blot analysis, suggesting that both cell lines secrete Cyr61. As a positive control for a cellular response to the BFA treatment, the 78 kDa glucose-regulated protein (Grp78) was investigated [39].

The induction of Cyr61 expression by EGF stimulation has been previously investigated mainly for cytoplasmic Cyr61. However, if Cyr61 acts as a ligand, e.g., for the integrins, the secreted Cyr61 is of particular relevance. For the investigation of the Cyr61 secretion, we selected MDA-MB-231 cells that are positive for the EGF receptor (Figure 4B). Moreover, we analysed the bone metastasis cell line B02 that was derived from MDA-MB-231. B02 displays strongly-elevated EGFR levels compared with MDA-MB-231 by Western blot analysis, such that the EGFR signal for MDA-MB-231 appears to be weak in this image. The comparison of the Cyr61 kinetics between MDA-MB-231 and B02 may also display specific changes that occur in the course of the colonization of the bone marrow.

First, we determined whether the cells respond to the EGF stimulation by the activation of the AKT pathway in MDA-MB-231 and B02 (Figure 4C). The response of the cells to EGF stimulation was determined by the increased phosphorylation of AKT on S473. The cytoplasmic Cyr61 levels remained unchanged compared with the untreated control after EGF stimulation in both cell lines. In contrast, the levels of extracellular Cyr61 were increased in EGF treated B02 and MDA-MB-231 cells (Figure 4D, Tables S2 and S3). Of note, after 0.25 h of EGF stimulation, the extracellular Cyr61 was not significantly elevated in B02 compared with the untreated control (*p* = 0.7801, Table S2), whereas for the MDA-MB-231 cells, significantly elevated Cyr61 levels were detected (*p* = 0.0077, Table S3). These data suggest that the bone-marrow–derived cell line responds with Cyr61 secretion slower after EGF stimulation than its parental cell line. Furthermore, these experiments show that the cells maintain their cytoplasmic Cyr61 levels even though the cells secrete considerable amounts of Cyr61 into the extracellular space, which is an important issue for CTC detection by Cyr61.

Due to the rapid response of MDA-MB-231 to EGF stimulation, we investigated how fast MDA-MB-231 are able to release Cyr61 into the cell culture supernatant. For this experiment, we replaced the cell culture medium with fresh medium, and determined the Cyr61 amount in the fresh medium (Figure S8A,B). After the medium exchange, we could even detect Cyr61 secretion after 5 min for MDA-MB-231 (*p* = 0.0009), and after 5 h, the MDA-MB-231 secreted a mass of approx. 2 pg Cyr61 per cell. In addition, we determined the time point at which BC-M1 begins the secretion after the medium replacement (Figure S8C,D). Here, we detected that BC-M1 secretes Cyr61 into the medium not until 1 h (*p* = 0.0036), and after 5 h the BC-M1 secretes a mass of approx. 1.6 pg Cyr61 per cell.

### 2.5. Cyr61 Expression in Clinical Breast Cancer Samples

**Figure 5:**
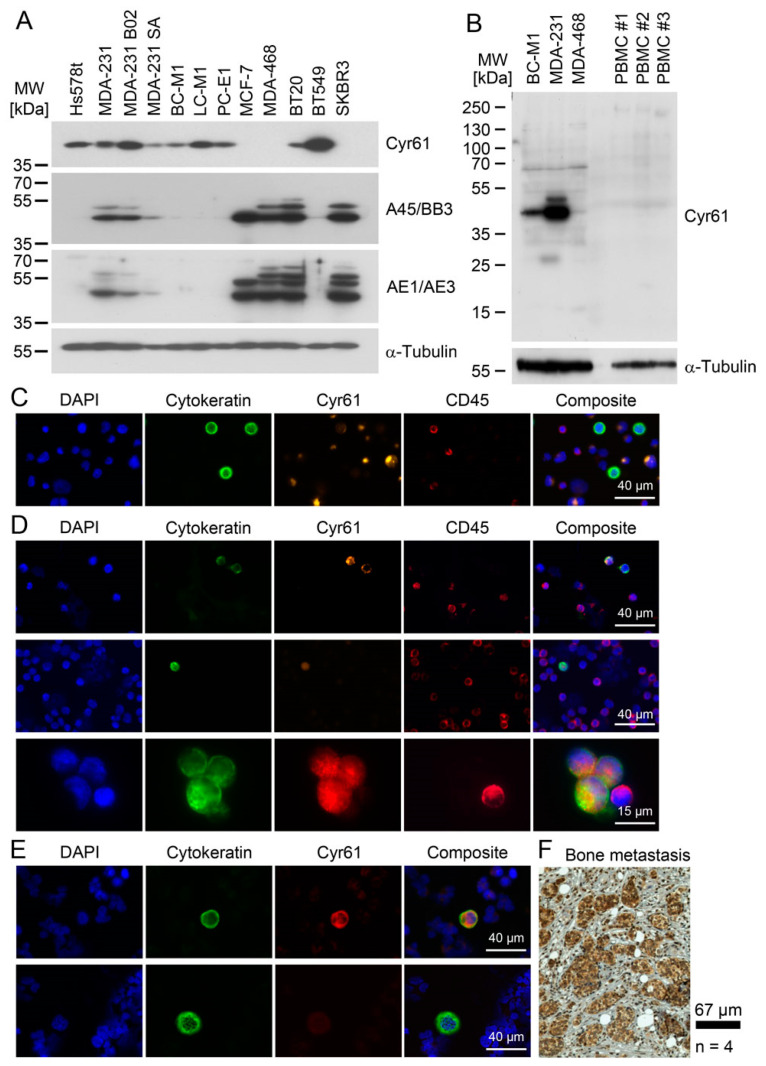
Cyr61 detection in breast cancer cells. (**A**) Comparison of the cytoplasmic Cyr61 levels with cytokeratin levels, as analysed by the pan-cytokeratin antibody cocktails A45/BB3 and AE1/AE3 by Western blot analysis. (**B**) A comparison of the Cyr61 levels in the peripheral blood mononuclear cells (PBMC) of healthy women with the Cyr61 levels in breast cancer cell lines. (**C**) Cyr61 detection in BC-M1 and MDA-MB-468 spiked into blood samples from healthy women by immunocytochemical double staining. (**D**) Cyr61 detection in CTC from the peripheral blood of breast cancer patients (details: Table 1). (**E**) Detection of Cyr61 in the DTC from the bone marrow of breast cancer patients. The upper row shows a Cyr61-positive DTC, and the bottom row shows a Cyr61-negative DTC. (**F**) An immunohistochemical Cyr61 detection in the bone metastases of breast cancer patients. (**C**–**E**) The composite images are overlays of the Cytokeratin, Cyr61, Dapi and CD45 (if applied) signals, n_biol_: 3 (**A**,**C**,**E**), 4 (**F**).

As we found that Cyr61 quickly responds to extracellular stimuli, we investigated whether Cyr61 might be a marker protein for CTC detection in patient samples. In a first step, we analysed a set of cell lines for the expression of Cyr61 and the most commonly used keratin-specific antibodies A45/BB3 and AE1/AE3 [40] (Figure 5A). The cell lines that were strongly positive for Cyr61 displayed a reduced expression of keratins, in which the epithelial/mesenchymal mixed phenotype cell line MDA-MB-231 displayed the expression both of Cyr61 and a restricted amount of keratins. In order to determine the correlation between keratin and Cyr61 in the cell lines, we compared the values for the antibody AE1/AE3 (Figures S9 and S10) with those of Cyr61 (Figure S1). For the statistical analysis we used Kendall’s Tau (k_τ_), and obtained a value of k_τ_ = −0.37799 and a *p*-value of 0.00124, showing that there is a negative correlation of Cyr61 and keratin in the cell lines, meaning that Cyr61 positive cells indeed display reduced keratin expression levels.

In the next step, we analyzed peripheral blood mononuclear cells (PBMC) from age-adapted healthy women (i.e., aged over 50 years) by Western blot analysis for the presence of Cyr61 (Figure 5B). Here, no detectable Cyr61 expression was observed by our assay, suggesting that PBMC express Cyr61 at a low level compared with breast cancer cells. Next, MDA-MB-468 and BC-M1 cells were spiked into PBMC from healthy women, and were then recaptured by a label-free technique (Parsortix, ANGLE PLC) that enriches tumor cells by their size and allows a reliable subsequent microscopic detection of Cyr61 (Figure 5C). The tumor cells were detected by the cytokeratin staining and counterstaining of leukocytes with CD45 antibodies, which ensured the assay’s specificity. These experiments confirmed that the Cyr61 levels in the PBMC were low compared with those in breast cancer cells, such that we expected only a low number of false positive events in the patient samples, which is an important prerequisite for the clinical application of a marker protein.

This assay was applied to peripheral blood samples freshly obtained from 35 breast cancer patients. In total, CTCs were detected in nine cases, and Cyr61-positive CTCs were found in four patients (Figure 5D; Table 1). Eight of these nine patients had a known positive CTC status, and four patients were positive for distant metastasis (TNM stage M_1_), while two patients were classified as being free of distant metastasis (M_0_), and detailed clinicopathological data were no longer available for three patients, because the initial diagnosis was performed in an external clinic. Cyr61 was present in 47 (43%) of the 109 detected CTCs. In some cases, Cyr61 was detected in CTC with low keratin staining intensity and a fragmented keratin staining pattern (Figure 5D upper panel), whereas the majority of the Cyr61-positive cells displayed a keratin staining pattern that spans through the cytoplasm (Figure 5D middle panel). In one case (Figure 5D bottom panel), we found a small cell cluster comprising three CTCs and one normal blood cell. Figure 5C exemplifies the quality of our assay: the CTCs were Cyr61 positive/CD45 negative, whereas the adjacent normal blood cell was Cyr61 negative/CD45 positive. Cyr61-positive/cytokeratin-negative cells were not detected by this assay.

We also applied the staining protocol for CTC detection on a small set of three bone marrow samples from breast cancer patients (Figure 5E), and detected both Cyr61-positive (Figure 5E upper panel) and Cyr61-negative DTCs (Figure 5E lower panel). As CTC might also originate from distant organs in patients with (occult) metastasis, we analysed whether the bone metastases express Cyr61 (Figure 5F). We detected Cyr61-positive tumor cells in all four overt bone metastasis specimens. Unfortunately, the primary tumors from the same patients were not available for comparative analysis.

## 3. Discussion

Even though recent developments in CTC research have provided new insights into cancer biology, and have opened novel areas of cancer diagnostics, new markers for the detection and characterization of CTCs are still needed [1]. Here, we introduced the stress-related protein Cyr61 as a potential new marker for CTCs. In order to detect Cyr61 in CTCs, sensitive multiplex immunostaining assays were developed, which allowed us to detect even breast tumor cells with low keratin levels. CTCs that weakly express keratins (as the standard CTC marker) are difficult to assess against background fluorescence; if these cells show a strong Cyr61 staining, it may help to reduce false negative results. We observed a marked heterogeneity of Cyr61 levels in CTCs both with regard to inter- and intra-patient variability, suggesting that CTCs have different abilities to respond to environmental stress.

We modelled, in cell lines, the response of Cyr61 to the pathophysiological parameters typically found in breast cancer patients in order to judge the usefulness of Cyr61 as a CTC/DTC detection marker in practice. These experiments revealed that the levels of Cyr61 are quite variable, depending on the specific stimulus, yet the overall Cyr61 expression is sufficient high in mCTC/mDTC for a confident detection in CTC/DTC from cancer patients.

For cytokeratin detection in CTC/DTC, we applied a sensitive and broad-range pan-cytokeratin antibody cocktail. We previously detected CK5 in the DTC cell lines on a low level by Western blot analysis [20,41]; therefore, we applied the keratin-specific antibody AE1/AE3 (together with the antibody C11), which detects CK5 with high sensitivity [42]. By this approach, we could detect both cancer cells with a strongly epithelial phenotype and cancer cells that show only a very low degree of cellular differentiation with low keratin expression [43]. This helped us to confirm the presence of Cyr61 in CTC from cancer patients, as our detection approach was suited to a CK5/Cyr61 positive phenotype like BC-M1, but could also detect CTCs with a high degree of epithelial differentiation. Interestingly, no Cyr61-positive/cytokeratin-negative cells were found, which might be an effect of our analytical approach emphasizing the detection of a tumor cell phenotype like BC-M1. Alternatively, breast cancer cells with a complete lack of all cytokeratin proteins might be very rare, or do not express Cyr61 [44].

Keratins are present in the cells as heteropolymers [45], which are difficult to disrupt to monomers by normal protein extraction methods. For the conversion to the keratin monomers that are then analysable by SDS-PAGE, the application of high-molar urea solutions is a suitable approach [46]. However, it cannot be excluded that the disruption to monomers is not complete, leading to a partial precipitation of keratin polymers during the SDS-PAGE. This might explain the low keratin signals for the DTC cell lines in some of our Western blots, even though we confirmed—by 2-D electrophoresis and mass spectrometry—that BC-M1 displays a reduced keratin expression pattern [41]. Therefore, a certain discrepancy in the measured keratin levels of the cell lines compared with those on CTCs of cancer patients might be due to keratin detection on CTCs by immunocytochemical detection, whereas for the cell lines, Western blot analysis was used.

We further interrogated the involvement of Cyr61 in stress responses that were relevant to disseminating tumor cells. After extravasation, CTCs may encounter a hypoxic microenvironment (e.g., in bone marrow), which affects the protein expression responding to this stress condition [47,48]. We therefore subjected the cell lines to 1% O_2_, which is the lower limit of the oxygen concentration in the bone marrow [19] and is sufficient to stabilize HIF-1α in most human cells [49]. In melanoma cells, a regulation of Cyr61 by HIF-1α was suggested, which is not mediated by the direct binding of HIF-1α to the Cyr61 promoter, but via c-Jun/AP-1 [27]. As it was also shown that high Cyr61 levels are associated with a more-aggressive breast cancer phenotype, this supports the idea of a selection process in vivo, which finally leads to aggressive phenotypes with high Cyr61 expression [27]. Our findings here support this idea, as all three investigated DTC cell lines were positive for Cyr61, and the bone metastasis sublines of MDA-MB-231—B02 and SA—also displayed high Cyr61 levels. Furthermore, we detected Cyr61 in the CTCs and overt bone metastases of breast cancer patients, closing the gap between the cell line experiments and the in situ findings in clinical specimens.

On the other hand, there might be an antagonistic regulative pathway that suppresses the Cyr61 levels under hypoxia. One candidate might be ErbB-2, which is strongly induced under hypoxia in MDA-MB-468 [28]. We observed here a downregulation of Cyr61 in this cell line. Cyr61 knock down did not affect the ErbB-2 levels in MDA-MB-231, suggesting that the ErbB-2 affects Cyr61 levels, but not vice versa.

Cyr61 is a matricellular protein present in both the cytoplasm and extracellular space [25]. In the extracellular region, it can act as a ligand for numerous receptors like the integrins. Unlike previous analyses by others, we simultaneously analyzed both the cytoplasmic and the secreted Cyr61 protein levels in order to obtain deeper insights into the biology of Cyr61. Moreover, the presence of cytoplasmic Cyr61 is crucial for the detection of CTC and DTC by Cyr61. Therefore, we investigated the possibility that tumor cells secrete large amounts of Cyr61 while maintaining cytoplasmic Cyr61 levels facilitating CTC and DTC detection even after secretion of large amounts of Cyr61 into the extracellular space.

As it has been reported before for endometrial cells that the *cyr61* mRNA can be induced by EGF stimulation via EGFR activation [26], we selected the EGFR-positive cell line MDA-MB-231 as a model. The activation of AKT after EGF treatment showed that the cells responded to the EGF stimulation, whereas the Cyr61 levels in the cytoplasm remained constant. In contrast, we observed a rapid increase in the secreted Cyr61 in the cell culture medium. Our initial hypothesis was that cytoplasmic Cyr61 serves as storages that can be quickly emptied upon the stimulation of the cells leading to Cyr61–weakly-positive cells that are difficult to detect in the circulation. Instead, we observed that the cytoplasmic Cyr61 levels remained constant, further suggesting that the increased total amount in the experiments (cytoplasmic + secreted Cyr61) results from the rapid novel synthesis of the Cyr61 proteins. The rapid induction of Cyr61 proteins might be due to its membership of the immediate-early genes that can be transcribed within minutes after activation [50]. From that point, a more complete view of the biological function of Cyr61 can be obtained when the secreted Cyr61 is analyzed, in particular, in experiments where no changes in the cytoplasmic levels were detected before.

We observed a similar cellular response of Cyr61 in the B02 cells, even though the EGFR levels in B02 were strongly elevated compared with MDA-MB-231, suggesting that the induction of Cyr61 secretion is not dependent on different EGFR levels in the cells. An interesting effect, which awaits future investigation, was the delayed Cyr61 secretion in B02 compared with MDA-MB-231. A similar effect was observed when we exchanged the cell culture medium for MDA-MB-231 and BC-M1. Future experiments might elucidate the reason for the different Cyr61 secretion kinetics between MDA-MB-231 and the bone marrow resident cells B02 and BC-M1. Finally, these experiments might imply that the amount of secreted Cyr61 is not necessarily proportional to its cytoplasmic form. This idea might be relevant for the interpretation of the Cyr61 levels that were obtained from solid tissue specimens [22,51].

Moreover, Cyr61 shows a complex multi-layered regulation, which may have implications for the interpretation of clinical data. In prostate cancer, patients showed a correlation of low Cyr61 protein levels with high recurrence rates [52]. Others reported, for breast cancer, that *cyr61* mRNA is frequently induced under hypoxia [53], whereas we found that the cytoplasmic Cyr61 protein is sometimes downregulated. The abrogation of the global mRNA translation under cell stress like hypoxia leads to mRNA accumulation, and to the blockading of the protein synthesis [47]. For *cyr61*, internal ribosome entry sites were detected, as well as the ability of *cyr61*-mRNA to undergo low cap-independent protein translation, allowing the synthesis of Cyr61 protein under cell stress [48,54,55]. Furthermore, alternative splice variants of the *cyr61* mRNA were detected in primary breast specimens that displayed the same level as the normal tissue [53]. Therefore, the correlation of a clinicopathological parameter or a biological response may depend on whether *cyr61*-mRNA, or cytoplasmic or secreted Cyr61 protein is being analysed, which may also explain the inconsistencies between published data on Cyr61.

One novel important aspect in cancer therapy is the use of immune checkpoint proteins. As PD-L1 was also detected in CTCs, it is an attractive therapeutic approach to target PD-L1 on CTCs [29,30]. Moreover, we previously found a manifest intra-patient and inter-patient heterogeneity in the PD-L1 expression in CTCs from patients with breast cancer [30]. Epithelial marker proteins like keratins are frequently used for the detection of PD-L1 in CTCs [30,56]. Here, we observed that mDTC lines with low keratin expression and mesenchymal attributes are strongly positive for PD-L1 and Cyr61. As Cyr61 is important for the induction of angiogenesis [57], such Cyr61/PD-L1 double-positive DTC may display a phenotype that is able to escape the immune system, and is able to establish novel tumor cell colonies at secondary sites. This hypothesis might explain the previous findings of other groups that Cyr61 is associated with a more-aggressive phenotype, and is important for metastatic progression in breast cancer [24]. Furthermore, it was reported that Cyr61 mediates resistance against estrogen [58], which might hamper endocrine therapies to prevent metastatic breast cancer.

Another regulative layer that affects the Cyr61 levels is the different half-life of the protein in the cytoplasm and the extracellular matrix. For the cytoplasmic Cyr61, a half-life of approximately 20–30 min, and for the extracellular Cyr61, a half-life of more than 24 h has been reported [50,59], which might have implications for its application as a CTC detection protein. CTC may experience a re-oxygenation pulse once the tumor cells are released from hypoxic tissue areas into the well-oxygenated blood circulation [28]. We observed that cytoplasmic Cyr61 is induced in BC-M1 under hypoxia, whereas the Cyr61 levels decreased to approximately 16% after 4 h of the reoxygenation of the cells, suggesting that Cyr61 is quickly degraded upon the reoxygenation of BC-M1. These properties can explain the rapid changes in Cyr61 protein concentrations following the in vitro re-oxygenation model used in his work to mimic the release to CTCs into the blood stream. Again, this effect appears to occur in a cell-line–specific manner, as we did not observe rapidly decreased Cyr61 levels in MDA-MB-231 and MDA-MB-468 after their reoxygenation.

As a consequence, DTC that re-enter the circulation may reduce their cytoplasmic Cyr61 levels during their passage to another secondary site. As the Cyr61 levels in BC-M1 are sufficiently high for confident immunocytochemical analysis, it might be conceivable that the subcellular location of Cyr61 changes during reoxygenation from nucleus-associated to proteasome-associated. If this is true, it would allow the discrimination of freshly-disseminated cells from those that have already spent more time in the circulation.

Recently, we showed that other proteins in CTCs that are therapeutic targets or biomarker proteins (e.g., PD-L1) alsoresponded rapidly to re-oxygenation [28]. This might be an important aspect to the consideration of whether these proteins are assessed in CTCs in clinical studies as surrogates of the molecular characteristics of tumor cells in primary or metastatic tissues. We previously observed in MDA-MB-231 and BC-M1 for PD-L1 a similar response to re-oxygenation as for Cyr61 in this work, suggesting a common regulative mechanism of these proteins.

Furthermore, in the Cyr61 knockdown cells, HIF-1α was downregulated, suggesting a regulative axis between Cyr61, c-Jun/AP-1, and HIF-1α, which needs to be further explored in future investigations. We found here that the loss of Cyr61 increases the apoptosis rates of cancer cells, which might be an interesting aspect in the context of PD-L1 detection and targeting in CTCs. Thus, future studies will show whether the Cyr61/PD-L1 double-positive phenotype might characterize a tumor cell population that might be of particular importance in breast cancer metastasis and its response to immunotherapy.

Taken together, Cyr61 might be suitable as a stress-related reporter for very rapid microenvironmental changes. Moreover, Cyr61 might serve as a novel marker for CTCs and DTCs with high plasticity in breast cancer. The remarkable regulation of Cyr61 might enable a perceptive readout of pathophysiological states, which may also provide future information on how to target disseminating cancer cells.

## 4. Materials and Methods

All of the cell line experiments were performed in biological replicates (n_biol_).

### 4.1. Patients

The human investigations were performed according to the Helsinki rules after approval was obtained by the ethics committee of the Medical Association of Hamburg (reference number PV5392). Written informed consent was obtained from all of the patients prior to any study-related procedures. Samples from women with breast cancer or healthy control persons treated at the University Medical Centre Hamburg-Eppendorf, Germany, were used. The blood from healthy persons was received from the Institute for Transfusion Medicine, University Medical Center Hamburg-Eppendorf. The fresh clinical samples from the breast cancer patients were drawn from breast cancer patients who were positive for distant metastases.

### 4.2. Cell Lines and Culture Conditions (Standard Cell Culture Condition)

The cultivation of the DTC cell lines was essentially performed as described [32]. A detailed overview of the generation, authentication, and properties of the DTC cell lines BC-M1 (obtained from the bone marrow of a breast cancer patient), LC-M1 (obtained from the bone marrow of a lung cancer patient), and PC-E1 (obtained from the bone marrow of a prostate cancer patient) were reported before [20]. The DTC cell lines were cultured at 37 °C in a humidified environment with 5% of carbon dioxide and 10% of oxygen. The oxygen concentration was adjusted by N_2_. The culture medium was RPMI 1640 supplemented with 10% fetal bovine serum, 2 mM L-glutamine, 10 mg/L insulin, 5.5 mg/L transferrin (all from Life Technologies, Darmstadt, Germany), 50 µg/L EGF (Miltenyi Biotec, Bergisch Gladbach, Germany) and 10 µg/L human basic fibroblast growth factor (b-FGF, Miltenyi Biotec). The bone metastatic sublines of MDA-MB-231, MDA-MB-231 SA [60], and MDA-MB-231 B02 [61] were cultivated in Dulbecco’s Modified Eagle Medium (DMEM) with 10% fetal calf serum (FCS) and 2 mM L-glutamine.

The cell lines by kind provision were: MDA-MB-231 B02 (Philippe Clézardin), MDA-MB-231 SA (Theresa A. Guise), Hs578t (Thomas Dittmar). MCF-7 (from ATCC, 2005; ATCC Cat# HTB-22), MDA-MB-231, MDA-MB-468 (DSMZ, Braunschweig, Germany, 09/2016; DSMZ Cat# ACC-738), BT20 (Cell Lines Service, Eppelheim, Germany, 2007; CLS Cat# 300130/p656_BT-20), BT549 (Cell Lines Service, 07/2008; CLS Cat# 300132/p770_BT-549), Hs578t, SCC25, Cal27, SKRBR3; they were cultivated in DMEM with 10% FCS and 2 mM L-glutamine (all from Life Technologies). The authenticated cell lines (last test) were MCF-7 (09/2017), MDA-MB-231 (02/2014), and Hs578t (09/2015). The authentication was performed by Multiplexion, Heidelberg, Germany by multiplex cell authentication (SNP-Profiling).

All of the cell lines were cultured at 37 °C in a humidified environment. The cell lines that were cultivated in RPMI were kept in the presence of 5% of CO_2_, and the cell lines that were cultured in DMEM were kept in the presence of 10% CO_2_. With the exception of the DTC cell lines, the remaining gas mixture was atmospheric air. These cell culture conditions are referred as to ‘standard cell culture conditions’ in this work. The cell lines were stored as cryo-cultures in liquid nitrogen, and the cells were temporarily resuscitated only for the experiments. After resuscitation, the cells were routinely tested for mycoplasmas using the VenorGeM Classic mycoplasma detection kit (Minerva Biolabs, Berlin, Germany, Cat. No. 11-1100). The mycoplasma test was performed after five passages (i.e., ±14 days), followed by the generation of fresh cyro-cultures if the cells were negative for mycoplasma. The mycoplasma-infected cells were expunged and the incubator was subjected to disinfestation. The experiments were performed from passage six to a maximum of 15 passages. The protein samples were generated within six months after the resuscitation of the cell lines.

### 4.3. Stable Isotope Labelling with Amino Acids in Cell Culture (SILAC) and Mass Spectrometry

The analysis of the protein expression profile of the breast cancer cell lines MDA-MB-468 and BC-M1 was performed as described [32], with the following modifications. The LC-MS/MS measurements were performed by injecting the samples onto a nano liquid chromatography system (Dionex UltiMate 3000 RSLCnano, Thermo Scientific, Bremen, Germany) coupled via electrospray-ionization (ESI) to a linear trap quadrupole (LTQ) orbitrap mass spectrometer (Orbitrap Fusion, Thermo Scientific, Bremen, Germany). The samples were loaded (3 µL/min) onto a trapping column (Acclaim PepMap nano trap column, C18, 3 µm, 150 mm × 0.075 mm, 100 Ǻ, Thermo Scientific, Bremen, Germany, buffer A: 0.1% FA in HPLC-H2O; buffer B: 0.1% FA in ACN) with 2% buffer B. After the sample loading, the trapping column was washed for 5 min with 2% buffer B (3 μL/min). The peptides were eluted (300 nL/min) onto the separation column (Acclaim PepMap 100, C18, 75 μm × 500 mm, 2 µm, 100 Ǻ, Thermo Scientific, Bremen, Germany) with a gradient of 2−30% B in 30 min. The spray was generated from a fused-silica emitter (I.D. 10 μm, New Objective, Woburn, MA, USA) at a capillary voltage of 1800 V. The mass spectrometric analysis was performed in the positive ion mode. The MS/MS analysis was carried out in data dependent acquisition mode (DDA) in the top speed mode using the following parameters: an HCD collision energy of 28%, an ion intensity threshold of 1 × 10^4^, and a quadrupole isolation width of 1.6 *m*/*z*. Every second, an MS scan was performed over an m/z range from 400–1500, with a resolution of 120,000 full width at half maximum (FWHM) at *m*/*z* 200 (transient length = 256 ms, maximum injection time = 50 ms, AGC target = 2 × 10^5^). The MS/MS spectra were recorded in the ion trap (scan-rate = 66 kDa/s, maximum injection time = 200 ms, AGC target = 1 × 10^4^). For the peptide and protein identification, Proteome Discoverer 2.0 with Sequest HT (Thermo Scientific, Bremen, Germany) and MaxQuant with Andromeda (version 1.5.2.8) was used. The MS/MS spectra were searched against a human SwissProt database (www.uniprot.org, downloaded 17 November 2017, 20,239 entries) and a contaminant database (298 entries). The searches were performed using the following parameters: the precursor mass tolerance was set to 10 ppm, and the fragment mass tolerance was set to 0.5 Da. Furthermore, two missed cleavages were allowed, as well as a carbamidomethylation on the cysteine residues as a fixed modification, an oxidation of the methionine residues, and a ^13^C_6_-label of the lysine and arginine residues as a variable modification. The peptides were identified with an FDR of 1%. The proteins were kept as correctly identified when at least one unique peptide was identified.

### 4.4. Special Cell Culture Conditions

Under special cell culture conditions, the culture conditions were the same as for the standard cell culture conditions, with the following modifications for each experiment. The cultivation of the cell lines in the presence of 1% O_2_ (hypoxia) was performed using the incubator Heracell 15 (Thermo Fisher Scientific, Waltham, MA, USA). The oxygen partial pressure was adjusted using N_2_. For the reoxygenation experiments, the cells were cultured under hypoxia for 14 days, followed by a reoxygenation pulse of 10% O_2_ for 4 h, which mimics the situation of CTCs in the blood stream. Ten percent oxygen is the average value for larger blood vessels, and the duration of 4 h is comparable to the life span of CTC in the blood circuit [35,36].

When the cell lines were cultured in a medium that contained no glucose (Glu^0^), the media “DMEM, no glucose” and “RPMI, no glucose” (both Life Technologies) were used. For the glucose starvation experiments in presence of 1% O_2_, both conditions were combined. For stabilization of HIF-1α, the cells were incubated with 150 µM cobalt chloride (Sigma-Aldrich, Munich, Germany) [62]. For the Brefeldin A (BFA) treatment, BFA was applied in order to analyse the Cyr61 secretion in cultured cells. The BFA was purchased from Merck (Calbiochem, Darmstadt, Germany, Cat. No. 203729-1MG), and was dissolved in DMSO in a concentration of 10 mg/mL (stock solution). The stock solution was diluted in 12 mL cell culture medium without FCS to a final concentration of 5 µg/mL for each 75 cm^2^ cell culture flask. The cells were incubated with BFA for 18 min under the standard cell culture conditions. For the control cells, DMSO without BFA was applied. The centrifugation steps were performed at 0 °C until the proteins of the culture medium were not dissolved in the lysis buffer (9.8 M urea, 15 mM EDTA, 30 mM Tris). The culture medium was collected and centrifuged at 2000× *g* for 5 min. The cell pellet of the detached cells was washed with 8 mL PBS, and the cells were lysed with lysis buffer and processed as described for the cell harvest for Western blot analysis. The supernatant (12 mL per cell culture flask) was concentrated by ultraspin centrifugal devices (Vivaspin 4, 10,000 Da MWCO, PES membrane; Sartorius-Stedim, Göttingen, Germany) to a volume of 200 µL. After addition of 3 mL lysis mix the supernatant was concentrated again to a volume of 200 µL. The cell pellet was harvested as described for the cell harvest for Western blot analysis. The three fractions (detached cells, cell lysate and supernatant) were subjected to Western blot analysis, as described. For the BFA experiments 50 µg of protein were applied for Western blot analysis. As BFA treatment leads to the activation of Grp78 [39], Grp78 induction served as a positive control. As the total protein amount for two of the three biological replicates of the MDA-MB-231 detached cells (untreated) was below 50 µg, a Western blot analysis lacking this sample is shown.

The EGF stimulation was performed as described [20], with the following modifications. For the EGF stimulation of MDA-231 and MDA-231 B02, the cells were cultured for 48 h in order to enrich the culture medium with secreted Cyr61 from the cells. Then, the culture medium was supplemented with 100 ng EGF per milliliter and incubated for the appropriate duration of time. As controls, cells were cultured without the addition of EGF to the culture medium in parallel. The cell culture supernatant was harvested, clarified by centrifugation, and analyzed for Cyr61 by an enzyme-linked immunosorbent assay, as described below.

### 4.5. Cell Harvest and Sample Procurement for Western Blot

The cells from the cell lines were washed three times with 37 °C prewarmed PBS and harvested in 300 µL lysis mix (9.8 M urea, 15 mM EDTA, 30 mM Tris) per 75 cm^2^ cell culture flask. The cell lysates were homogenized on ice by ultrasonic treatment using the ultrasonic device UP50H (Hielscher, Teltow, Germany) by three identical steps (amplitude 100%; 10 s) and incubated at room temperature for 1 h, followed by centrifugation (15,000× *g* at room temperature for 5 min) and the collection of the supernatant.

The purified PBMCs were washed with 1 mL of PBS. The cells were then incubated in the lysis mix and homogenized by ultrasonic treatment. Subsequently, the proteins were purified by precipitation using 600 µL precipitant (component of the 2-D Quant Kit, GE Healthcare, Uppsala, Sweden) per 250 µL of the sample, and a co-precipitant (component of the 2-D Quant Kit) in the same amount [41]. The purified proteins were dissolved in 100 µL 9.8 M urea and solubilized for 1 h at room temperature. The protein concentration was determined using the Pierce BCA Protein Assay Kit (Pierce, Rockford, IL, USA) according to the manufacturer’s instructions, and using BSA as the standard. The samples were stored at −80 °C. The sample quality and the quality of the BCA-test results were confirmed by colloidal Coomassie-stained SDS gels. The staining procedure was performed according to Neuhoff [63].

### 4.6. SDS-PAGE and Western Blot

The SDS-PAGE and Western blot analysis were performed as described [32], with the following modifications. For the Western blot analysis, 20 µg protein or 40 µg protein per sample were applied. After the SDS-PAGE, the proteins were transferred to Immobilon-P^SQ^ membranes (Millipore GmbH, Schwalbach, Germany). The proteins were transferred by tank blot using the mini VE vertical electrophoresis system equipped with tank blot transfer units (GE Healthcare, Uppsala, Sweden). The bands were visualized using the Signal Fire ECL Reagent (Cell Signaling Technology, Danvers, MA, USA) and X-ray films (Agfa HealthCare, Mortsel, Belgium) in accordance with the manufacturer’s instructions. The X-ray films were digitized using a GS-700 imaging densitometer (Bio-Rad, Hercules, CA, USA). The densitometric analysis was performed using Quantity one software (Bio-Rad). Each reaction was performed in biological triplicates. The applied antibodies are listed in Table 2.

The membranes were stripped using the following stripping buffer: 7.56 g Tris, 20 g SDS, 7.8 g 2-mercaptoethanol, adjustment to pH 9.5 using HCl, and H_2_O ad 1 l. Prior to its use, 0.1 g DTT was freshly added to 25 mL stripping buffer. The membranes were incubated at room temperature with gentle agitation for 45 min. After washing with tris-buffered saline with 0.1% Tween 20 (TBST), the membranes were incubated with blocking buffer for one hour, and another primary antibody was applied. The cleavage of the disulphide bridges by the reducing agents is very efficient at alkaline pH values, such that the removal of antibodies can be performed under mild conditions, allowing the multiple rehybridization of the membrane with antibodies.

In the case of the quantitative analysis of X-ray films, the values of different biological experiments were combined. First, the signals of the protein of interest were normalized to their associated alpha-tubulin values obtained from the same polyvinylidene difluoride (PVDF) membrane to level out the potential differences in the protein loading and transfer (intra-gel comparison). For the inter-gel comparison, the values were normalized by setting one value of one single experiment to 100 a. u. (usually the standard cell culture condition). After that, the values from the different experimental runs that were not set to 100 a. u. were compared. The antibodies used are specified in Table 2.

For the correlation analysis between Cyr61 and keratin, we used Kendall’s Tau (k_τ_) and the software OriginPro (version 9.6.5.169, OriginLab Corporation, Northampton, USA).

### 4.7. Lentivirus-Mediated Knockdown of Cyr61 Gene Expression

The lentiviral pLKO.1 shRNA vectors targeted against human Cyr61 (*cyr61)* were designed by The RNAi Consortium (Broad Institute of Harvard and Massachusetts Institute of Technology; GE Dharmacon, Uppsala, Sweden; Cat. No. RHS4533-EG3491 [64]). The used vectors contained the following sequences:

Cyr61#1: TRCN0000118097 (Clone ID);
CCGGGCAAACAGAAATCAGGTGTTTCTCGAGAAACACCTGATTTCTGTTTGCTTTTTG (Forward Oligo Sequence);
AATTCAAAAAGCAAACAGAAATCAGGTGTTTCTCGAGAAACACCTGATTTCTGTTTGC (Reverse Oligo Sequence).
Cyr61#2: TRCN0000118098 (Clone ID);
CCGGCGCATCCTATACAACCCTTTACTCGAGTAAAGGGTTGTATAGGATGCGTTTTTG (Forward Oligo Sequence);
AATTCAAAAACGCATCCTATACAACCCTTTACTCGAGTAAAGGGTTGTATAGGATGCG (Reverse Oligo Sequence).
Cyr61#3: TRCN0000118099 (Clone ID);
CCGGCCGAACCAGTCAGGTTTACTTCTCGAGAAGTAAACCTGACTGGTTCGGTTTTTG (Forward Oligo Sequence);
AATTCAAAAACCGAACCAGTCAGGTTTACTTCTCGAGAAGTAAACCTGACTGGTTCGG (Reverse Oligo Sequence).
Cyr61#4: TRCN0000118100 (Clone ID);
CCGGCCCTTCTACAGGCTGTTCAATCTCGAGATTGAACAGCCTGTAGAAGGGTTTTTG (Forward Oligo Sequence);
AATTCAAAAACCCTTCTACAGGCTGTTCAATCTCGAGATTGAACAGCCTGTAGAAGGG (Reverse Oligo Sequence).
Cyr61#5: TRCN0000118101 (Clone ID);
CCGGCGAACCAGTCAGGTTTACTTACTCGAGTAAGTAAACCTGACTGGTTCGTTTTTG (Forward Oligo Sequence);
AATTCAAAAACGAACCAGTCAGGTTTACTTACTCGAGTAAGTAAACCTGACTGGTTCG (Reverse Oligo Sequence).

The pLKO.1 vectors harboring a scrambled non-target shRNA sequence (Addgene, Cambridge, MA, USA) served as a negative control (non-target control). The lentiviruses were made by transfection of HEK-293T packaging cells with these constructs by the use of a three-plasmid system, as described [65]. The supernatants were harvested, sterile filtered, and used to infect MDA-MB-231 cells in the presence of 8 µg/mL polybrene (Sigma-Aldrich) as the final concentration, and with a multiplicity of infection (MOI) of 1. The pooled stable transfectants were established using an optimal final puromycin concentration of 0.5 µg/mL. These experiments were performed under the standard culture conditions for MDA-MB-231. After seven days, the knockdown efficiency was analysed by Western blot analysis and an enzyme-linked immunosorbent assay. Cyr61#4 and Cyr61#5 showed no detectable Cyr61 knockdown in MDA-MB-231 by Western blot analysis, and were not analysed further. The transfectants of Cyr61#2 and Cyr61#3 were used for the research of their biological functions with apoptosis and proliferation assays under standard cell culture conditions, and under hypoxia (1% O_2_). The photomicrographs were taken using an Axiovert 25 inverted microscope (Carl Zeiss AG, Oberkochen, Germany).

### 4.8. Enzyme-Linked Immunosorbent Assay (ELISA)

The cell lysates and culture supernatants were clarified by centrifugation at 2500× *g* for 15 min. The recombinant human Cyr61 protein was purchased from Abnova (Taipei, Taiwan).

For the coating of the wells, the anti-Cyr61 antibody H2 (Santa Cruz Biotechnology, Santa Cruz, CA, USA) was applied. The antibody was diluted 1:250 in DMEM with 10% FCS. The plate was incubated at 4 °C overnight, with gentle agitation. In order to remove the unbound antibody, the wells were washed with 100 µL volume three times. The first time, the wells were washed with PBS, followed by two steps using PBS with 0.02% Tween 20 (Roth, Karlsruhe, Germany). Next, the unspecific binding was blocked with blocking buffer (5% nonfat dry milk [Roth], in PBS with 0.02% Tween) using 100 µL blocking buffer per well. Next, three washing steps using 100 µL volume each, as described, were performed. For the incubation with cell culture supernatant, 2.5 µL of the sample was diluted in 97.5 µL DMEM with 10% FCS. This was followed by three washing steps, as described. Next, the anti-Cyr61 antibody H78 (Santa Cruz Biotechnology) was added to the wells. The anti-Cyr61 antibody was diluted 1:500 in DMEM with 10% FCS. This was followed by three washing steps. For the detection of Cyr61, a polyclonal goat anti-rabbit immunoglobulin antibody coupled with horseradish peroxidase (DAKO, Glostrup, Denmark) was diluted 1:250 with blocking buffer. The reaction was incubated at room temperature for one hour, with gentle agitation. This was followed by three washing steps. Next, 100 µL 3,3′,5,5′-tetramethylbenzidine (TMB) one-component horseradish peroxidase (HRP) microwell substrate (Bethyl Laboratories, Montgomery, PA, USA) was added to each well. The incubation was carried out whilst protected from light at room temperature for 15 min. The reaction was stopped by the addition of 100 µL Stop Solution for TMB Substrates (Immunochemistry Technologies, Bloomington, IN, USA) and incubation in the dark with gentle agitation for 15 min. The extinction at 450/620 nm was detected using the ELISA reader NanoQuant infinite M200 pro (Tecan, Männedorf, Switzerland). The OD values were converted to Cyr61 concentrations using recombinant and purified Cyr61 protein as a standard.

### 4.9. Proliferation and Apoptosis Assays

For the analysis of the cell proliferation and apoptosis, MDA-MB-231 Cyr61 knock down cells (Cyr61#2 and Cyr61#3) and the corresponding non-target control cells were applied. The cell proliferation was analysed by the incorporation of 5-bromo-2′-deoxyuridine (BrdU) into the DNA of the proliferating cells using the BrdU Cell Proliferation Assay Kit (Cell Signaling Technology). The cells were plated in a concentration of 30,000 cells per well in 96-well TC plates, Standard, F (Sarstedt, Nümbrecht, Germany) and cultured either under the standard cell culture conditions or under 1% O_2_ for 72 h. Subsequently, the BrdU solution was added to the cells and incubated on the cells for 24 h. Next, 100 µL of the Fixing/Denaturing Solution was added to each well and incubated for 30 min, followed by the incubation of 100 µL of the detection antibody solution per well for 1 h. After the washing of the wells, the HRP-conjugated secondary antibody solution was applied to the wells and incubated at room temperature for 30 min. For the detection, 100 µL 3,3′,5,5′-tetramethylbenzidine (TMB) substrate was applied to each well and incubated at room temperature whilst protected from light. After the addition of the stop solution, the absorbance was detected at 450 nm using the ELISA reader NanoQuant infinite M200 pro.

The apoptosis of the cells was analysed by annexin V staining using APC Annexin V (BioLegend, San Diego, CA, USA). The cells were cultured under the standard culture conditions, or under 1% O_2_. After that, the cells were washed twice with Cell Staining Buffer (BioLegend) and resuspended in Annexin V Binding Buffer (BioLegend) to a final concentration of 1 × 10^6^ cells/mL. The cell suspension was supplemented with 5 µL APC Annexin V per 100 µL of the cell suspension. After incubation for 15 min at room temperature, protected from light, 400 µL Annexin V Binding Buffer was added to each sample and analysed by flow cytometry (FACSCanto II, Franklin Lakes, NJ, USA).

### 4.10. Size Based CTC Enrichment

We applied a marker-independent separation device (Parsortix, ANGLE PLC, Surrey, UK) for the tumor cell enrichment. The Parsortix system uses a micro-fluidic technology in the form of a disposable cassette (Cell separation cassette cc3R, Parsortix) to capture CTCs out of the blood from cancer patients. In total, 4 ml blood was collected into BD Vacutainers (BD Belliver Industrial Estate, Plymouth, UK). The blood was pumped automatically through the cassette. The cassette enriches CTCs based on their larger size (≥10 µm) compared to other blood components. In order to reduce the background noise, the cassette was automatically washed with PBS (Life Technologies). The isolated tumor cells were harvested, cytospun on a slide (SuperFrost/Plus, Glaswarenfabrik Karl Hecht KG, Sondheim, Germany), and stained, as described below. For the cell line spiking experiments with blood from healthy persons, fresh blood was received from the Institute for Transfusion Medicine, University Medical Center Hamburg-Eppendorf. The clinical samples from breast cancer patients were drawn from the Department of Gynecology.

### 4.11. Purification of the Bone Marrow Specimens and Peripheral Blood Samples

Bone marrow was aspirated bilaterally from both the anterior and posterior iliac crests (10 mL/site) from breast cancer patients or healthy volunteers. The following procedures were accomplished under sterile conditions. The bone marrow aspirates were washed in Hanks’ Balanced Salt solution (HBSS) (Biochrom AG, Berlin, Germany), diluted in Dulbecco’s phosphate-buffered saline (DPBS) (Life Technologies), and separated by density centrifugation using Ficoll Paque Plus (GE Healthcare, Munich, Germany). Mononuclear cells were collected from the interphase layer and washed twice in phosphate buffered saline (PBS) with 10% fetal calf serum (Biological Industries, Kibbutz Beit Haemek, Israel). The cytospins were prepared by centrifuging the bone marrow mononuclear cells onto glass slides (Superfrost plus, Glaswarenfabrik Karl Hecht KG, Sondheim, Germany; 7 × 10^5^ mononuclear cells per slide). The slides were air-dried overnight and stored at −80 °C.

For the experiments using peripheral blood from healthy individuals, the PBMC were separated by Ficoll density centrifugation. The obtained PBMC were collected from the interphase layer, washed with PBS, and either applied for the spiking experiments or analyzed by Western blot analysis. For the spiking experiments, the cell lines were spiked into the blood or bone marrow from healthy individuals and processed as described.

### 4.12. Immunocytochemical Cyr61 Detection in Cell Lines, Blood and Bone Marrow Samples

The cell lines were spiked into the blood or bone marrow samples of healthy control persons. The immunocytochemical double staining was performed by the application of the anti-Cyr61 antibody (H78) in combination with a Cytokeratin-specific antibody cocktail. The Cytokeratin antibody cocktail consisted of anti-pan-keratin antibodies (mouse monoclonal, clones AE1/AE3; Merck) and anti-pan-keratin antibody C11 (mouse monoclonal; Cell Signaling Technology Cat# 4545). The direct conjugates of AE1/AE3-Alexa Fluor488 (Thermo Fisher Scientific Cat# 53-9003-82) and C11-Alexa Fluor488 (Cell Signaling Technology Cat# 4523) were used when stated for the individual experiments. The detection of normal blood cells was performed using the anti-CD45 antibody coupled with Alexa Fluor 647 (BioLegend, Cat# 304018), when stated.

The slides were thawed 30 min prior to their incubation. The cells were permeabilized for 10 min with 1% Triton X-100 in PBS. A washing step was followed by the blocking of unspecific binding using AB-Serum (Biotest, Dreieich, Germany, Cat. No. 805 135) (10% in PBS) for 20 min. The primary antibody against Cyr61 was added in a 1:50 dilution and incubated at room temperature for 1 h. The slides were washed again three times with PBS, and Alexa 546 or Alexa 532 goat anti-rabbit secondary antibody (Molecular Probes, Eugene, OR, USA; Thermo Fisher Scientific Cat# A-11035, Thermo Fisher Scientific Cat# A-11009) was applied. After three washing steps with PBS, the Cytokeratin specific antibodies were applied and incubated for 60 min. For the AE1/AE3 C11 antibody cocktail, the dilution was 1:700 for the AE1/AE3, and the C11 was diluted 1:200. The residual Cytokeratin-specific antibodies were removed by three washing steps with PBS. If unconjugated Cytokeratin specific antibodies were applied, the secondary Alexa 488 rabbit anti-mouse fluorochrome antibody (Molecular Probes; Molecular Probes Cat# A-11059) was added in a 1:200 dilution in 10% AB-Serum and incubated for 45 min. After another washing step (3 × with PBS), the slides were covered with Vectashield Mounting Medium containing Dapi (Vector Laboratories, Burlingame, USA). The staining controls were run in parallel, using dilution media instead of the primary and secondary antibody. The slides were evaluated manually using the microscope Axioplan 2 (Carl Zeiss AG, Oberkochen, Germany).

### 4.13. Cyr61 Immunohistochemical Staining

For the immunohistochemical (IHC) staining, the anti-Cyr61 antibody (H78) rabbit polyclonal (Santa Cruz Biotechnology, Santa Cruz, CA, USA) was applied. This antibody was applied previously by another working group for TMA staining in prostate cancer [52]. Paraffin-embedded specimens on microscope slides of breast cancer patients were applied. The paraffin wax was removed by the incubation of the specimen at 60 °C for two hours, followed by the incubation of the samples twice in xylene for 10 min each. In order to remove the xylene, the slides were incubated in 99% ethanol, followed by incubation in 96% ethanol and in 80% ethanol. After a brief washing step in water, the samples were autoclaved at 120 °C in citrate buffer (pH 6.0) for 5 min. Thereafter, the sections were rinsed with TBST for 5 min. A peroxidase treatment was performed using the Dako REAL Peroxidase-Blocking Solution (DAKO, Cat. No. S202386-2) for five minutes. After a brief washing step with TBST, the anti-Cyr61 antibody was applied. The antibody was used in a 1:750 dilution using the Dako Antibody Diluent (DAKO) and incubated at 4 °C overnight. Subsequently, three 3-min washing steps with TBST were performed. For the detection of the primary antibody, labelled polymer-HRP and the secondary antibody was used from the DAKO REAL Detection system Peroxidase/DAB (DAKO #K5001) according to the manufacturer’s instructions. For the chromogenic detection, 3,3’-diaminobenzidine (DAB) was applied. After a brief washing step, the nuclei were visualized by haematoxylin staining (Merck, Darmstadt, Germany). For the preservation of the specimen, Eukitt mounting medium (Kindler, Freiburg, Germany) was used.

## 5. Conclusions

In conclusion, the present work assessed the potential role of a new CTC biomarker that appears to be a sensor of changes in micro-environmental conditions and might support the survival of disseminating tumor cells under stress, e.g., hypoxia. Future studies implementing Cyr61 assessment into clinical studies using CTCs for the prediction of clinical outcomes in cancer patients will provide information on the clinical relevance of this protein as potential indicator of an aggressive CTC subset.

## Figures and Tables

**Table 1:** CTC and Cyr61 detection rates in the blood samples from breast cancer patients. ER: estrogen receptor alpha status; PR: progesterone receptor status.

**All Patients**	**Analysed Samples**		**CTC Positive (Cytokeratin)**		**Cyr61 Positive CTC**		**Distant Metastasis**	**ER**	**PR**	**ErbB-2**
	***n***	**%**	***n***	**%**	***n***	**%**				
	35	100	9	25.7	4	11.4				
CTC positive patients	Cytokeratin positive CTC		Cytokeratin/Cyr61 positive CTC		Cytokeratin positive/Cyr61 negative CTC					
Patient 1	11		9		2		M1	+	+	−
Patient 2	3		0		3		n/a ^a^	n/a	n/a	n/a
Patient 3	8		1		7		M0	−	−	−
Patient 4	1		0		1		M1	+	+	−
Patient 5	39		7		32		M1	+	+	−
Patient 6	1		0		1		n/a	n/a	n/a	n/a
Patient 7	5		0		5		n/a	n/a	n/a	n/a
Patient 8	1		0		1		M1	+	+	−
Patient 9	40		30		10		M0	+	+	−
Sum CTC (%)	109 (100)		47 (43.1)		62 (56.9)					

^a^ Data not available, because the initial diagnosis was performed in an external clinic.

**Table 2:** Antibodies and conditions used for the Western blot analysis.

Antibody	Supplier	Clonality	Catalog Number	Dilution	Other
Anti-ErbB-2	Abcam, Cambridge, UK	mouse monoclonal (clone CB11)	ab8054	1:500	
Anti-vimentin	BD Pharmingen, Erembodegem, Belgium	mouse monoclonal (clone RV202)	550513	1:5000	
Anti-Integrin αv	BD biosciences, Heidelberg, Germany	mouse monoclonal (clone 21/CD51)	611012	1:500	
Anti-AKT	Cell Signaling Technology, Danvers, USA	rabbit polyclonal	9272	1:5000	
Anti-phospho AKT (Ser473)	Cell Signaling Technology, Danvers, USA	rabbit monoclonal (clone 193H12)	4058	1:500	
Anti-α-Tubulin	Cell Signaling Technology, Danvers, USA	rabbit monoclonal (clone 11H10)	2125	1:10,000	
Anti-BiP	Cell Signaling Technology, Danvers, USA	rabbit monoclonal (clone C50B12)	3177	1:1000	BiP is a synonym for Grp78
Anti-Cyr61	Cell Signaling Technology, Danvers, USA	rabbit polyclonal	11952	1:1000	
Anti-HIF-1α	Cell Signaling Technology, Danvers, USA	rabbit monoclonal (clone D2U3T)	14179	1:1000	
Anti-Integrin β3	Cell Signaling Technology, Danvers, USA	rabbit monoclonal (clone D7X3P)	13166	1:2000	
Anti-PD-L1	Cell Signaling Technology, Danvers, USA	rabbit monoclonal (clone E1L3N)	13684	1:2000	
Anti-E-cadherin	Epitomics, Burlingame, CA, USA	rabbit monoclonal (clone EP700Y)	ab40772	1:20,000	
Anti-Cytokeratin Epithelial Antibody	Merck (Chemicon), Darmstadt, Germany	mouse monoclonal (clone AE1)	MAB1612	1:10,000	AE1 and AE3 were combined in 1:1 ratio for Western Blot
Anti-Cytokeratin Epithelial Antibody	Merck (Chemicon), Darmstadt, Germany	mouse monoclonal (clone AE3)	MAB1611	1:10,000	
Anti-pan-cytokeratin Antibody	Micromet, Munich, Germany	mouse monoclonal (clone A45/BB3)	n/a	1:10,000	
Anti-N-Cadherin	Novus Biologicals, Littleton, CO, USA	rabbit monoclonal (clone EPR1792Y)	NB110-55645	1:10,000	
Anti-Cyr61 (H78)	Santa Cruz Biotechnology, Santa Cruz, CA, USA	rabbit polyclonal	sc-13100	1:1000 (MDA-MB-468, MCF-7, PBMC) or 1:10,000 (all other cell lines)	
Anti-Cyr61 (H2)	Santa Cruz Biotechnology, Santa Cruz, CA, USA	mouse monoclonal	sc-271217	1:1000 (MDA-MB-468, MCF-7) or 1:10,000 (all other cell lines)	

## Data Availability

The data presented in this study are available in the article and supplementary material.

## Supplementary Materials

The following are available online at https://www.mdpi.com/2072-6694/13/3/563/s1, Figure S1: Quantitative analysis of the Western Blots shown in Figure 1C of the main text. Figure S2: Scatterplot for Figure 2A of the main text. Figure S3: Scatterplot for Figure 2E of the main text. Figure S4: Scatterplot for Figure 3A of the main text. Figure S5: Scatterplot for Figure 3C of the main text. Figure S6: Scatterplot for Figure 3E of the main text. Figure S7: Scatterplot for Figure 4D of the main text. Figure S8: Cyr61 secretion rates in MDA-MB-231 and BC-M1. Figure S9: Quantitative analysis of the Western Blots shown in Figure 5A of the main text. Figure S10. Uncropped Western blot analysis x-ray films for Figure 5A of the main text. Table S1: (separate Excel sheet). Cyr61 identification and quantification details. Table S2: Analysis of the response of Cyr61 in MDA-MB-231 B02 after EGF stimulation. Table S3: Analysis of the response of Cyr61 in MDA-MB-231 after EGF stimulation. All of the uncropped Western blot analysis gels are on the end of Supporting Information.

## References

[1] Bardelli A., Pantel K. (2017). Liquid Biopsies, What We Do Not Know (Yet). Cancer Cell.

[2] Pantel K., Alix-Panabieres C. (2019). Liquid biopsy and minimal residual disease—Latest advances and implications for cure. Nat. Rev. Clin. Oncol..

[3] Keller L., Pantel K. (2019). Unravelling tumour heterogeneity by single-cell profiling of circulating tumour cells. Nat. Rev. Cancer.

[4] Pantel K., Alix-Panabieres C., Riethdorf S. (2009). Cancer micrometastases. Nat. Rev. Clin. Oncol..

[5] Braun S., Vogl F.D., Naume B., Janni W., Osborne M.P., Coombes R.C., Schlimok G., Diel I.J., Gerber B., Gebauer G. (2005). A pooled analysis of bone marrow micrometastasis in breast cancer. N. Engl. J. Med..

[6] Janni W., Vogl F.D., Wiedswang G., Synnestvedt M., Fehm T., Juckstock J., Borgen E., Rack B., Braun S., Sommer H. (2011). Persistence of disseminated tumor cells in the bone marrow of breast cancer patients predicts increased risk for relapse—A European pooled analysis. Clin. Cancer Res..

[7] Braun S., Kentenich C., Janni W., Hepp F., de Waal J., Willgeroth F., Sommer H., Pantel K. (2000). Lack of effect of adjuvant chemotherapy on the elimination of single dormant tumor cells in bone marrow of high-risk breast cancer patients. J. Clin. Oncol..

[8] Cristofanilli M., Pierga J.Y., Reuben J., Rademaker A., Davis A.A., Peeters D.J., Fehm T., Nole F., Gisbert-Criado R., Mavroudis D. (2019). The clinical use of circulating tumor cells (CTCs) enumeration for staging of metastatic breast cancer (MBC): International expert consensus paper. Crit. Rev. Oncol. Hematol..

[9] Naume B., Synnestvedt M., Falk R.S., Wiedswang G., Weyde K., Risberg T., Kersten C., Mjaaland I., Vindi L., Sommer H.H. (2014). Clinical outcome with correlation to disseminated tumor cell (DTC) status after DTC-guided secondary adjuvant treatment with docetaxel in early breast cancer. J. Clin. Oncol..

[10] Riethdorf S., Wikman H., Pantel K. (2008). Review: Biological relevance of disseminated tumor cells in cancer patients. Int. J. Cancer.

[11] Kang Y., Pantel K. (2013). Tumor cell dissemination: Emerging biological insights from animal models and cancer patients. Cancer Cell.

[12] Tam W.L., Weinberg R.A. (2013). The epigenetics of epithelial-mesenchymal plasticity in cancer. Nat. Med..

[13] Bartkowiak K., Riethdorf S., Pantel K. (2011). The Interrelating Dynamics of Hypoxic Tumor Microenvironments and Cancer Cell Phenotypes in Cancer Metastasis. Cancer Microenviron..

[14] Yu M., Bardia A., Wittner B.S., Stott S.L., Smas M.E., Ting D.T., Isakoff S.J., Ciciliano J.C., Wells M.N., Shah A.M. (2013). Circulating breast tumor cells exhibit dynamic changes in epithelial and mesenchymal composition. Science.

[15] O’Flaherty L., Wikman H., Pantel K. (2017). Biology and clinical significance of circulating tumor cell subpopulations in lung cancer. Transl. Lung Cancer Res..

[16] Ameri K., Luong R., Zhang H., Powell A.A., Montgomery K.D., Espinosa I., Bouley D.M., Harris A.L., Jeffrey S.S. (2010). Circulating tumour cells demonstrate an altered response to hypoxia and an aggressive phenotype. Br. J. Cancer.

[17] Salnikov A.V., Liu L., Platen M., Gladkich J., Salnikova O., Ryschich E., Mattern J., Moldenhauer G., Werner J., Schemmer P. (2012). Hypoxia induces EMT in low and highly aggressive pancreatic tumor cells but only cells with cancer stem cell characteristics acquire pronounced migratory potential. PLoS ONE.

[18] Parmar K., Mauch P., Vergilio J.A., Sackstein R., Down J.D. (2007). Distribution of hematopoietic stem cells in the bone marrow according to regional hypoxia. Proc. Natl. Acad. Sci. USA.

[19] Spencer J.A., Ferraro F., Roussakis E., Klein A., Wu J., Runnels J.M., Zaher W., Mortensen L.J., Alt C., Turcotte R. (2014). Direct measurement of local oxygen concentration in the bone marrow of live animals. Nature.

[20] Bartkowiak K., Effenberger K.E., Harder S., Andreas A., Buck F., Peter-Katalinic J., Pantel K., Brandt B.H. (2010). Discovery of a novel unfolded protein response phenotype of cancer stem/progenitor cells from the bone marrow of breast cancer patients. J. Proteome Res..

[21] Menendez J.A., Mehmi I., Griggs D.W., Lupu R. (2003). The angiogenic factor CYR61 in breast cancer: Molecular pathology and therapeutic perspectives. Endocr. Relat. Cancer.

[22] Tsai M.S., Hornby A.E., Lakins J., Lupu R. (2000). Expression and function of CYR61, an angiogenic factor, in breast cancer cell lines and tumor biopsies. Cancer Res..

[23] Jiang W.G., Watkins G., Fodstad O., Douglas-Jones A., Mokbel K., Mansel R.E. (2004). Differential expression of the CCN family members Cyr61, CTGF and Nov in human breast cancer. Endocr. Relat. Cancer.

[24] Huang Y.T., Lan Q., Lorusso G., Duffey N., Ruegg C. (2017). The matricellular protein CYR61 promotes breast cancer lung metastasis by facilitating tumor cell extravasation and suppressing anoikis. Oncotarget.

[25] Lau L.F. (2011). CCN1/CYR61: The very model of a modern matricellular protein. Cell Mol. Life Sci..

[26] Klein R., Stiller S., Gashaw I. (2012). Epidermal growth factor upregulates endometrial CYR61 expression via activation of the JAK2/STAT3 pathway. Reprod Fertil. Dev..

[27] Kunz M., Moeller S., Koczan D., Lorenz P., Wenger R.H., Glocker M.O., Thiesen H.J., Gross G., Ibrahim S.M. (2003). Mechanisms of hypoxic gene regulation of angiogenesis factor Cyr61 in melanoma cells. J. Biol. Chem..

[28] Bartkowiak K., Koch C., Gartner S., Andreas A., Gorges T.M., Pantel K. (2020). In Vitro Modeling of Reoxygenation Effects on mRNA and Protein Levels in Hypoxic Tumor Cells upon Entry into the Bloodstream. Cells.

[29] Hofman P., Heeke S., Alix-Panabieres C., Pantel K. (2019). Liquid biopsy in the era of immuno-oncology: Is it ready for prime-time use for cancer patients?. Ann. Oncol..

[30] Mazel M., Jacot W., Pantel K., Bartkowiak K., Topart D., Cayrefourcq L., Rossille D., Maudelonde T., Fest T., Alix-Panabieres C. (2015). Frequent expression of PD-L1 on circulating breast cancer cells. Mol. Oncol..

[31] Grabinski N., Bartkowiak K., Grupp K., Brandt B., Pantel K., Jucker M. (2011). Distinct functional roles of Akt isoforms for proliferation, survival, migration and EGF-mediated signalling in lung cancer derived disseminated tumor cells. Cell. Signal..

[32] Bartkowiak K., Kwiatkowski M., Buck F., Gorges T.M., Nilse L., Assmann V., Andreas A., Muller V., Wikman H., Riethdorf S. (2015). Disseminated Tumor Cells Persist in the Bone Marrow of Breast Cancer Patients through Sustained Activation of the Unfolded Protein Response. Cancer Res..

[33] Putz E., Witter K., Offner S., Stosiek P., Zippelius A., Johnson J., Zahn R., Riethmuller G., Pantel K. (1999). Phenotypic characteristics of cell lines derived from disseminated cancer cells in bone marrow of patients with solid epithelial tumors: Establishment of working models for human micrometastases. Cancer Res..

[34] Mohme M., Riethdorf S., Pantel K. (2016). Circulating and disseminated tumour cells—Mechanisms of immune surveillance and escape. Nat. Rev. Clin. Oncol..

[35] Ward J.P. (2008). Oxygen sensors in context. Biochim. Biophys. Acta.

[36] Meng S., Tripathy D., Frenkel E.P., Shete S., Naftalis E.Z., Huth J.F., Beitsch P.D., Leitch M., Hoover S., Euhus D. (2004). Circulating tumor cells in patients with breast cancer dormancy. Clin. Cancer Res..

[37] Yuan Y., Hilliard G., Ferguson T., Millhorn D.E. (2003). Cobalt inhibits the interaction between hypoxia-inducible factor-alpha and von Hippel-Lindau protein by direct binding to hypoxia-inducible factor-alpha. J. Biol. Chem..

[38] Vellon L., Menendez J.A., Lupu R. (2005). AlphaVbeta3 integrin regulates heregulin (HRG)-induced cell proliferation and survival in breast cancer. Oncogene.

[39] Dery M.A., Jodoin J., Ursini-Siegel J., Aleynikova O., Ferrario C., Hassan S., Basik M., Leblanc A.C. (2013). Endoplasmic reticulum stress induces PRNP prion protein gene expression in breast cancer. Breast Cancer Res..

[40] Vincent-Salomon A., Bidard F.C., Pierga J.Y. (2008). Bone marrow micrometastasis in breast cancer: Review of detection methods, prognostic impact and biological issues. J. Clin. Pathol..

[41] Bartkowiak K., Wieczorek M., Buck F., Harder S., Moldenhauer J., Effenberger K.E., Pantel K., Peter-Katalinic J., Brandt B.H. (2009). Two-dimensional differential gel electrophoresis of a cell line derived from a breast cancer micrometastasis revealed a stem/progenitor cell protein profile. J. Proteome Res..

[42] Effenberger K.E., Borgen E., Eulenburg C.Z., Bartkowiak K., Grosser A., Synnestvedt M., Kaaresen R., Brandt B., Nesland J.M., Pantel K. (2011). Detection and clinical relevance of early disseminated breast cancer cells depend on their cytokeratin expression pattern. Breast Cancer Res. Treat..

[43] Korsching E., Packeisen J., Agelopoulos K., Eisenacher M., Voss R., Isola J., van Diest P.J., Brandt B., Boecker W., Buerger H. (2002). Cytogenetic alterations and cytokeratin expression patterns in breast cancer: Integrating a new model of breast differentiation into cytogenetic pathways of breast carcinogenesis. Lab. Investig..

[44] Willipinski-Stapelfeldt B., Riethdorf S., Assmann V., Woelfle U., Rau T., Sauter G., Heukeshoven J., Pantel K. (2005). Changes in Cytoskeletal Protein Composition Indicative of an Epithelial-Mesenchymal Transition in Human Micrometastatic and Primary Breast Carcinoma Cells. Clin. Cancer Res..

[45] Karantza V. (2011). Keratins in health and cancer: More than mere epithelial cell markers. Oncogene.

[46] Eichner R., Kahn M. (1990). Differential extraction of keratin subunits and filaments from normal human epidermis. J. Cell Biol..

[47] Wouters B.G., Koritzinsky M. (2008). Hypoxia signalling through mTOR and the unfolded protein response in cancer. Nat. Rev. Cancer.

[48] Baird S.D., Turcotte M., Korneluk R.G., Holcik M. (2006). Searching for IRES. RNA.

[49] Denko N.C. (2008). Hypoxia, HIF1 and glucose metabolism in the solid tumour. Nat. Rev. Cancer.

[50] Yang G.P., Lau L.F. (1991). Cyr61, product of a growth factor-inducible immediate early gene, is associated with the extracellular matrix and the cell surface. Cell Growth Differ..

[51] Chen P.P., Li W.J., Wang Y., Zhao S., Li D.Y., Feng L.Y., Shi X.L., Koeffler H.P., Tong X.J., Xie D. (2007). Expression of Cyr61, CTGF, and WISP-1 correlates with clinical features of lung cancer. PLoS ONE.

[52] D’Antonio K.B., Schultz L., Albadine R., Mondul A.M., Platz E.A., Netto G.J., Getzenberg R.H. (2010). Decreased expression of Cyr61 is associated with prostate cancer recurrence after surgical treatment. Clin. Cancer Res..

[53] Hirschfeld M., zur Hausen A., Bettendorf H., Jager M., Stickeler E. (2009). Alternative splicing of Cyr61 is regulated by hypoxia and significantly changed in breast cancer. Cancer Res..

[54] Chiluiza D., Bargo S., Callahan R., Rhoads R.E. (2011). Expression of truncated eukaryotic initiation factor 3e (eIF3e) resulting from integration of mouse mammary tumor virus (MMTV) causes a shift from cap-dependent to cap-independent translation. J. Biol. Chem..

[55] Johannes G., Carter M.S., Eisen M.B., Brown P.O., Sarnow P. (1999). Identification of eukaryotic mRNAs that are translated at reduced cap binding complex eIF4F concentrations using a cDNA microarray. Proc. Natl. Acad. Sci. USA.

[56] Strati A., Koutsodontis G., Papaxoinis G., Angelidis I., Zavridou M., Economopoulou P., Kotsantis I., Avgeris M., Mazel M., Perisanidis C. (2017). Prognostic significance of PD-L1 expression on circulating tumor cells in patients with head and neck squamous cell carcinoma. Ann. Oncol..

[57] Brigstock D.R. (2002). Regulation of angiogenesis and endothelial cell function by connective tissue growth factor (CTGF) and cysteine-rich 61 (CYR61). Angiogenesis.

[58] Tsai M.S., Bogart D.F., Castaneda J.M., Li P., Lupu R. (2002). Cyr61 promotes breast tumorigenesis and cancer progression. Oncogene.

[59] O’Brien T.P., Yang G.P., Sanders L., Lau L.F. (1990). Expression of cyr61, a growth factor-inducible immediate-early gene. Mol. Cell. Biol..

[60] Pollari S., Kakonen S.M., Edgren H., Wolf M., Kohonen P., Sara H., Guise T., Nees M., Kallioniemi O. (2012). Enhanced serine production by bone metastatic breast cancer cells stimulates osteoclastogenesis. Breast Cancer Res. Treat..

[61] Peyruchaud O., Winding B., Pecheur I., Serre C.M., Delmas P., Clezardin P. (2001). Early detection of bone metastases in a murine model using fluorescent human breast cancer cells: Application to the use of the bisphosphonate zoledronic acid in the treatment of osteolytic lesions. J. Bone Miner. Res..

[62] Fu O.Y., Hou M.F., Yang S.F., Huang S.C., Lee W.Y. (2009). Cobalt chloride-induced hypoxia modulates the invasive potential and matrix metalloproteinases of primary and metastatic breast cancer cells. Anticancer. Res..

[63] Neuhoff V., Arold N., Taube D., Ehrhardt W. (1988). Improved staining of proteins in polyacrylamide gels including isoelectric focusing gels with clear background at nanogram sensitivity using Coomassie Brilliant Blue G-250 and R-250. Electrophoresis.

[64] Moffat J., Grueneberg D.A., Yang X., Kim S.Y., Kloepfer A.M., Hinkle G., Piqani B., Eisenhaure T.M., Luo B., Grenier J.K. (2006). A lentiviral RNAi library for human and mouse genes applied to an arrayed viral high-content screen. Cell.

[65] Werner S., Frey S., Riethdorf S., Schulze C., Alawi M., Kling L., Vafaizadeh V., Sauter G., Terracciano L., Schumacher U. (2013). Dual roles of the transcription factor grainyhead-like 2 (GRHL2) in breast cancer. J. Biol. Chem..

