# Supplementary material for: Cysteine-Rich Angiogenic Inducer 61: Pro-Survival Function and Role as a Biomarker for Disseminating Breast Cancer Cells
Source: Cancers (Basel). 2021 Feb 2;13(3):563. doi: 10.3390/cancers13030563 (PMC7867178; doi:10.3390/cancers13030563)
Supplement: Supplementary file 1 [file cancers-13-00563-s001.zip › cancers-1065436-supplementary/Supplementary files/cancers-980384 R1 Supporting Information.pdf]

cancers

MDPI

Article

Cysteine-rich angiogenic inducer 61: Pro-survival function and role as biomarker for disseminating breast cancer cells

Kai Bartkowiak, Isabel Heidrich, Marcel Kwiatkowski, Tobias M Gorges, Antje Andreas, Maria Geffken, Karl Verpoort, Volkmar Müller, Hartmut Schlüter, Klaus Pantel

Supporting Information

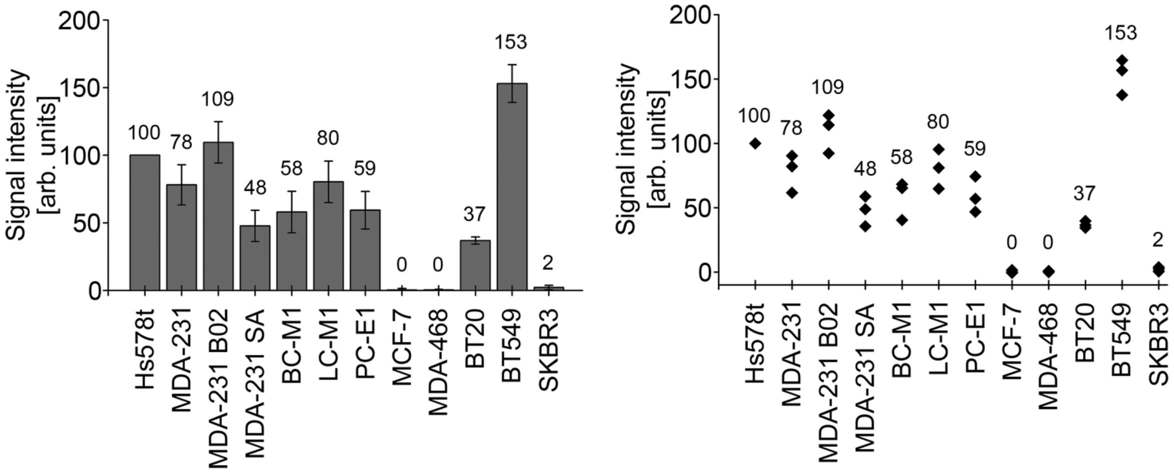

Figure S1. Quantitative analysis of the Western Blots shown in Fig. 1C of the main text. For each experiment, the signal intensities of Cyr61 were normalized to the values of alpha-Tubulin. For linkage of the biological replicates, the values of Hs578t were set to 100 a. u. for each experiment. Three biological replicates were analyzed for each cell line and each data point represents one single measurement value. Numbers represents the average values. Left: Display of the average values and the standard deviation (vertical error bars). Right: Display of the single measurement values. The cells were cultured under standard conditions.

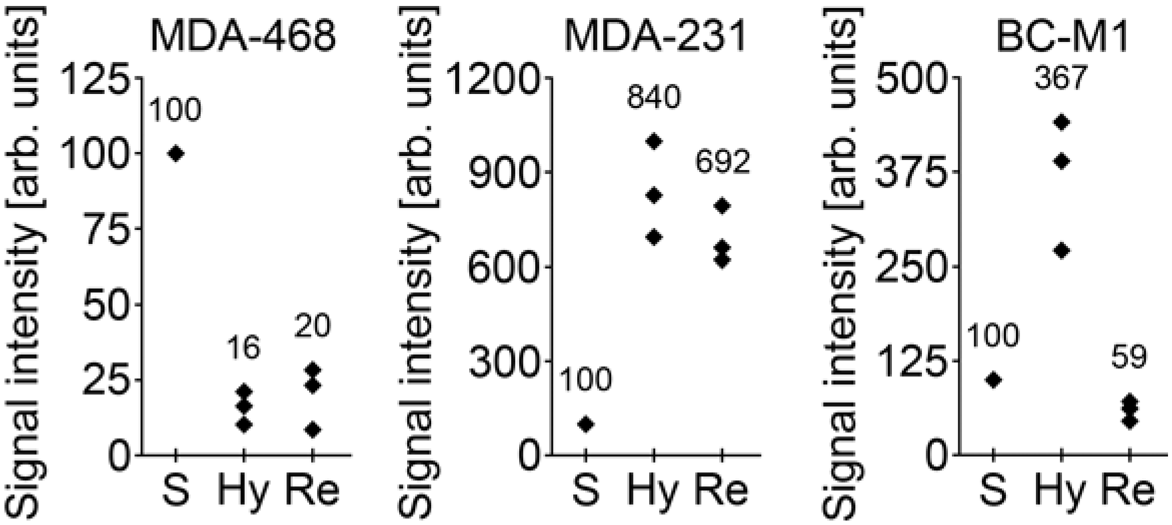

Figure S2. Scatterplot for Figure 2A of the main text.

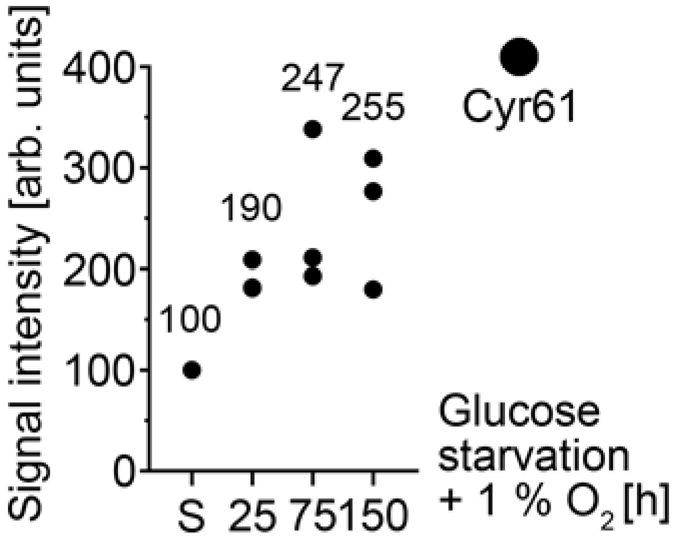

Figure S3. Scatterplot for Figure 2E of the main text.

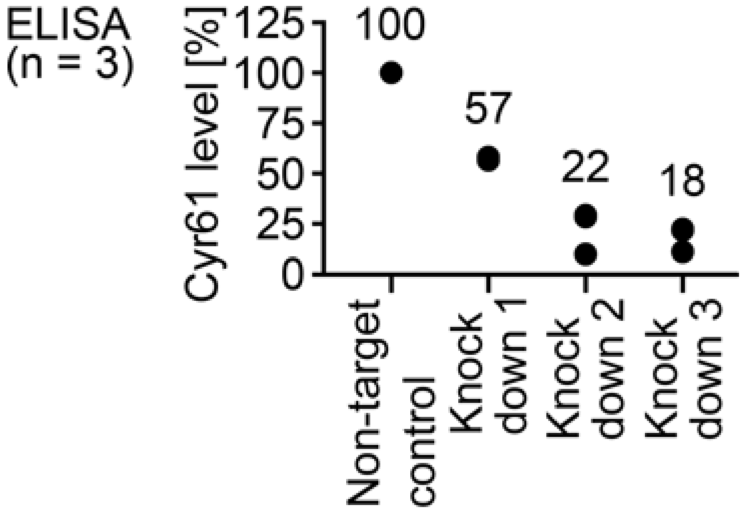

Figure S4. Scatterplot for Figure 3A of the main text.

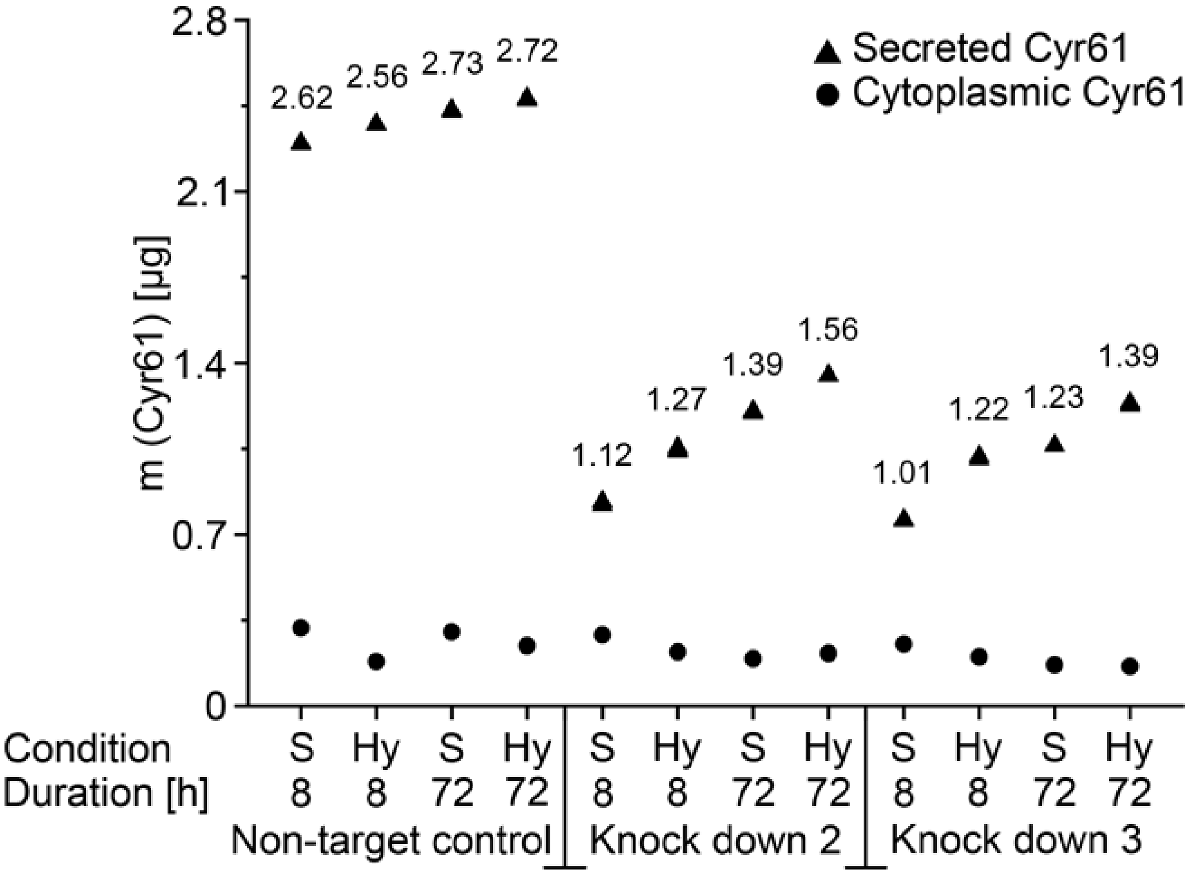

Figure S5. Scatterplot for Figure 3C of the main text.

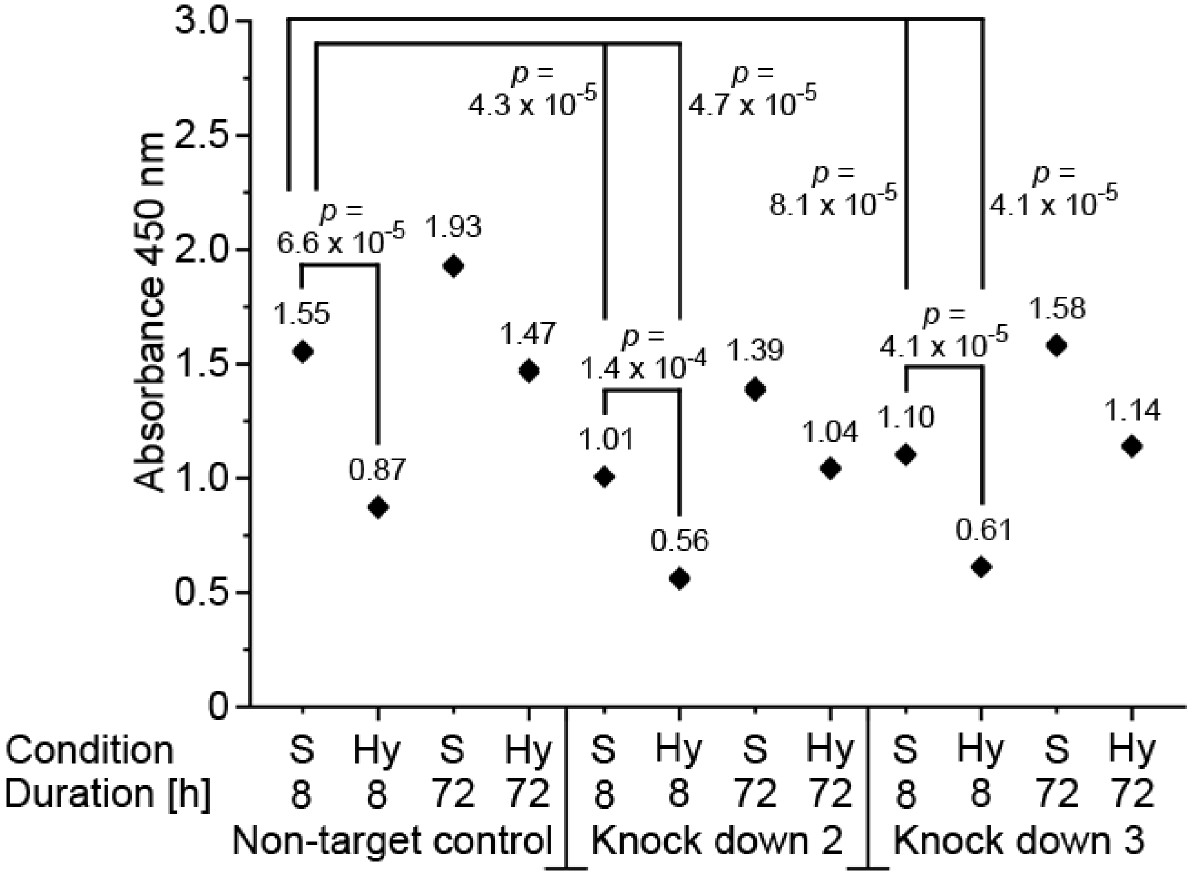

Figure S6. Scatterplot for Figure 3E of the main text.

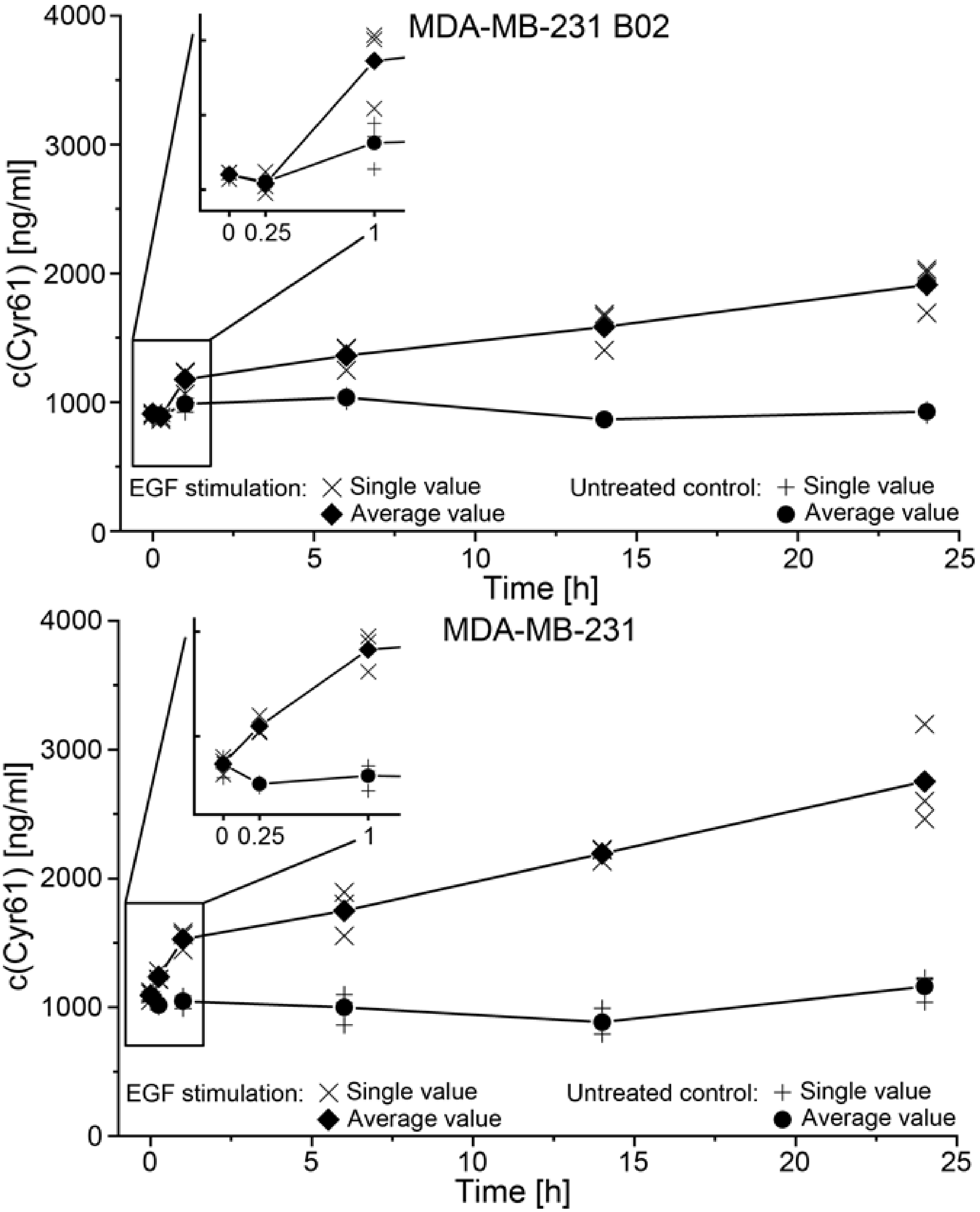

Figure S7. Scatterplot for Figure 4D of the main text.

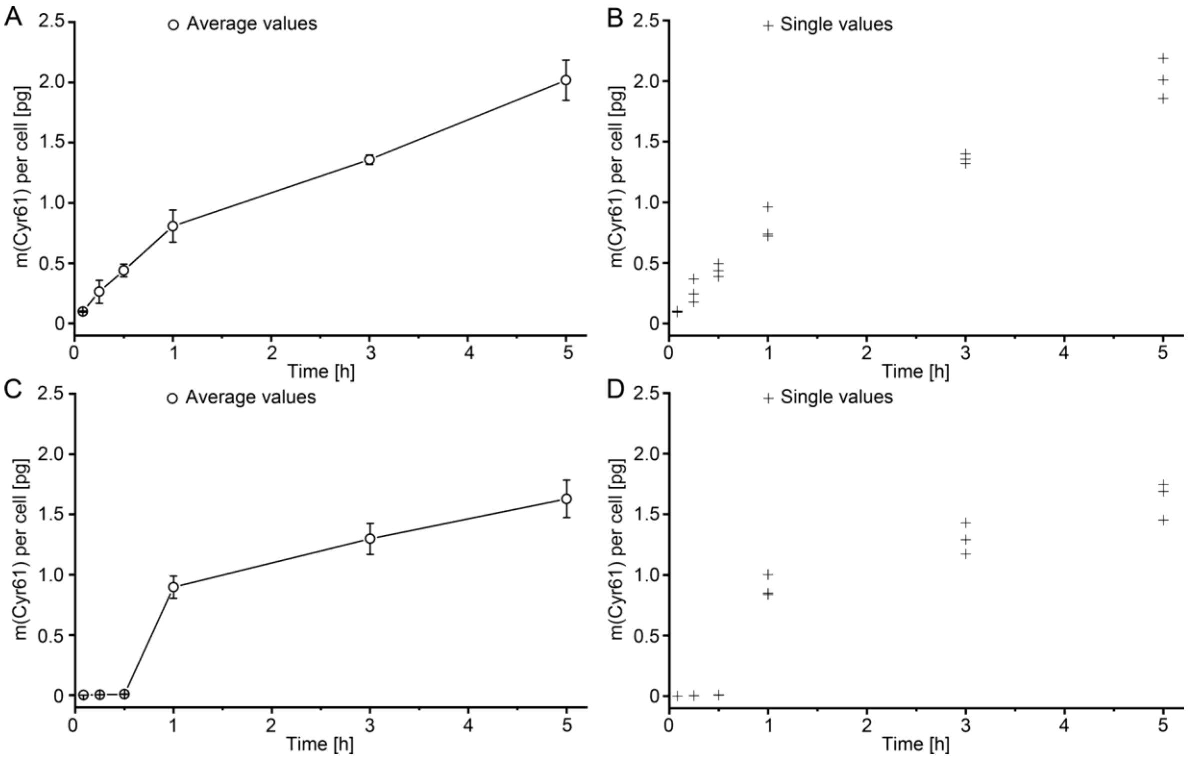

Figure S8. Cyr61 secretion in MDA-MB-231 (A, B) and BC-M1 (C, D) after replacement of the cell culture medium at t = 0 h. The total amount of the Cyr61 mass in the cell culture supernatant was determined by ELISA. After determination of the cell number, the mass of Cyr61 per cell [pg] was calculated. The experiment was performed under standard cell culture conditions. For each time point, three biological samples were analyzed for each cell line. Figures A and C display the average values and the standard deviation (vertical error bars) and figures B and D show the individual measurement values.

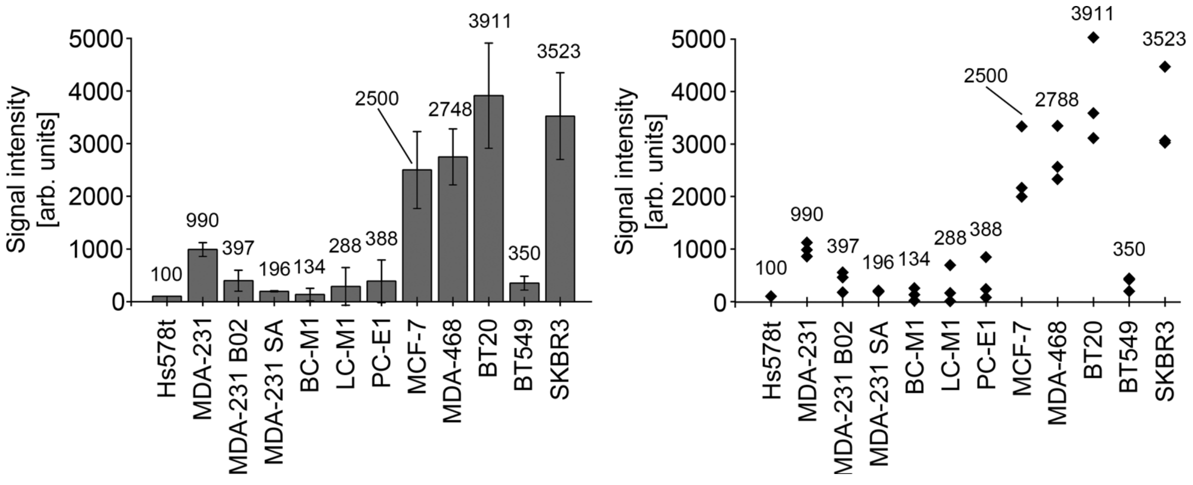

Figure S9. Quantitative analysis of the Western Blots shown in Fig. 5A of the main text. For each experiment, the signal intensities of keratin (detected by the antibody AE1/AE3) were normalized to the values of alpha-Tubulin. For linkage of the biological replicates, the values of Hs578t were set to 100 a. u. for each experiment. Three biological replicates were analyzed for each cell line and each data point represents one single measurement value. Numbers represents the average values. Left: Display of the average values and the standard deviation (vertical error bars). Right: Display of the single measurement values. The cells were cultured under standard conditions.

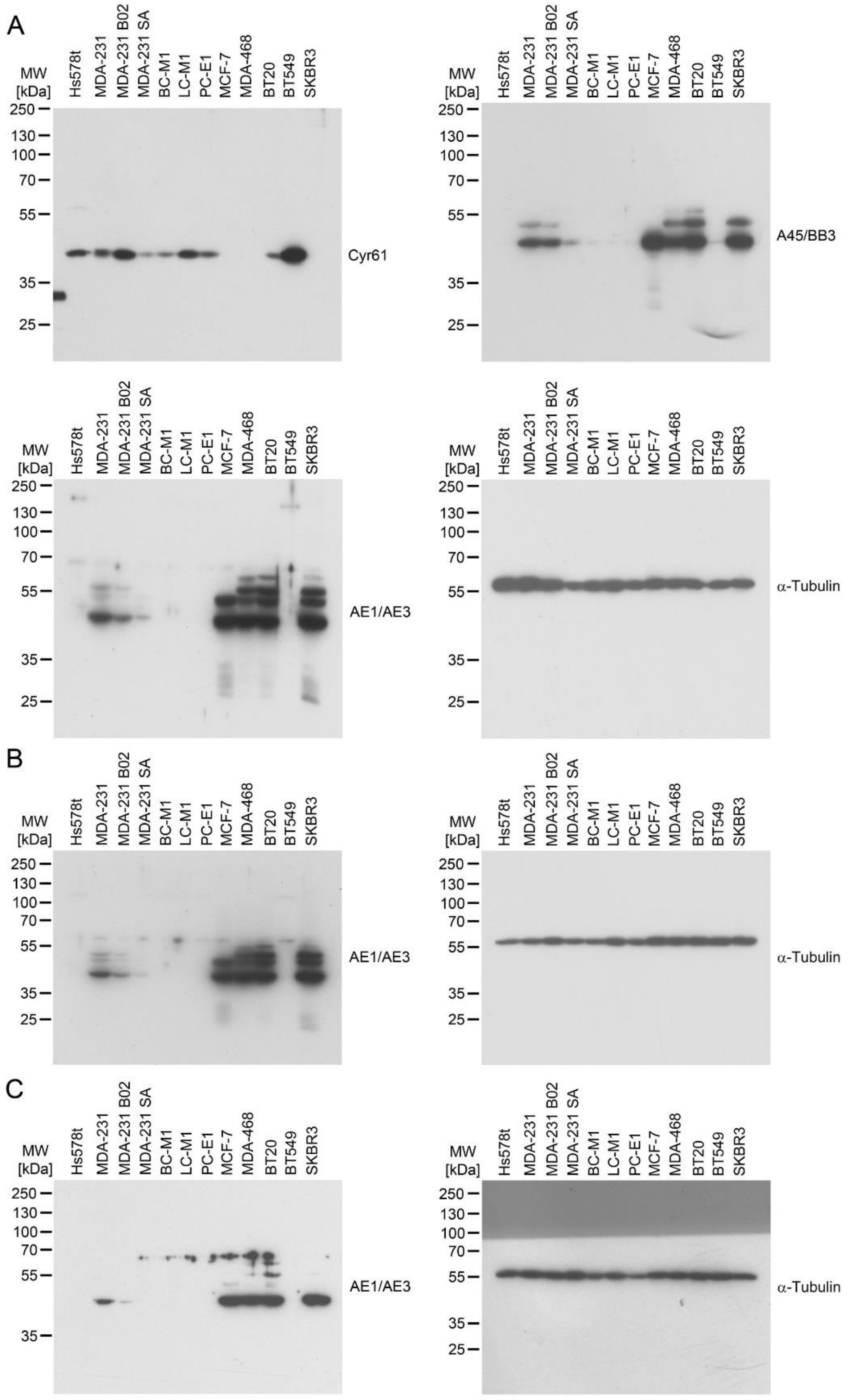

Figure S10. A: Uncropped Western Blot x-ray films for Figure 5A of the main text. B: Uncropped Western Blot x-ray film images for keratin detected by the antibody AE1/AE3 and the corresponding loading control alpha-Tubulin. The x-ray film images for AE1/AE3 and the corresponding loading controls from Figure S10 A-C were used for the quantitative analysis of the Western Blots shown in Fig. S9. The signals at 60-70 kDa are the basic keratins (e. g. CK5/6) that are difficult to display by Western Blot due to protein precipitation during the SDS-PAGE gel run. The cells were cultured under standard conditions.

Table S2: Analysis of the response of Cyr61 in MDA-MB-231 B02 after EGF stimulation

Time [min]	B02 untreated (c_{Cyr61} [ng/ml])		B02 EGF stimulation (c_{Cyr61} [ng/ml])		t-test (untreated vs. EGF stimulation)
	Arithmetic mean	Standard deviation	Arithmetic mean	Standard deviation	
0	910	9	910	8	-
15	893	9	889	18	0.7801
60	984	55	1176	83	0.0176
360	1035	25	1361	85	0.0160
840	865	1	1584	134	0.0153
1440	925	24	1911	162	0.0094

Table S3: Analysis of the response of Cyr61 in MDA-MB-231 after EGF stimulation

Time [min]	MDA-MB-231 untreated (c_{Cyr61} [ng/ml])		MDA-MB-231 EGF stimulation (c_{Cyr61} [ng/ml])		t-test (untreated vs. EGF stimulation)
	Arithmetic mean	Standard deviation	Arithmetic mean	Standard deviation	
0	1093	46	1093	35	-
15	1017	11	1238	37	0.0077
60	1048	50	1530	73	0.0187
360	1000	123	1750	175	0.0050
840	885	101	2194	51	0.0010
1440	1162	106	2754	391	0.0180

Uncropped Western Blot x-ray film images

For Figure 1C:

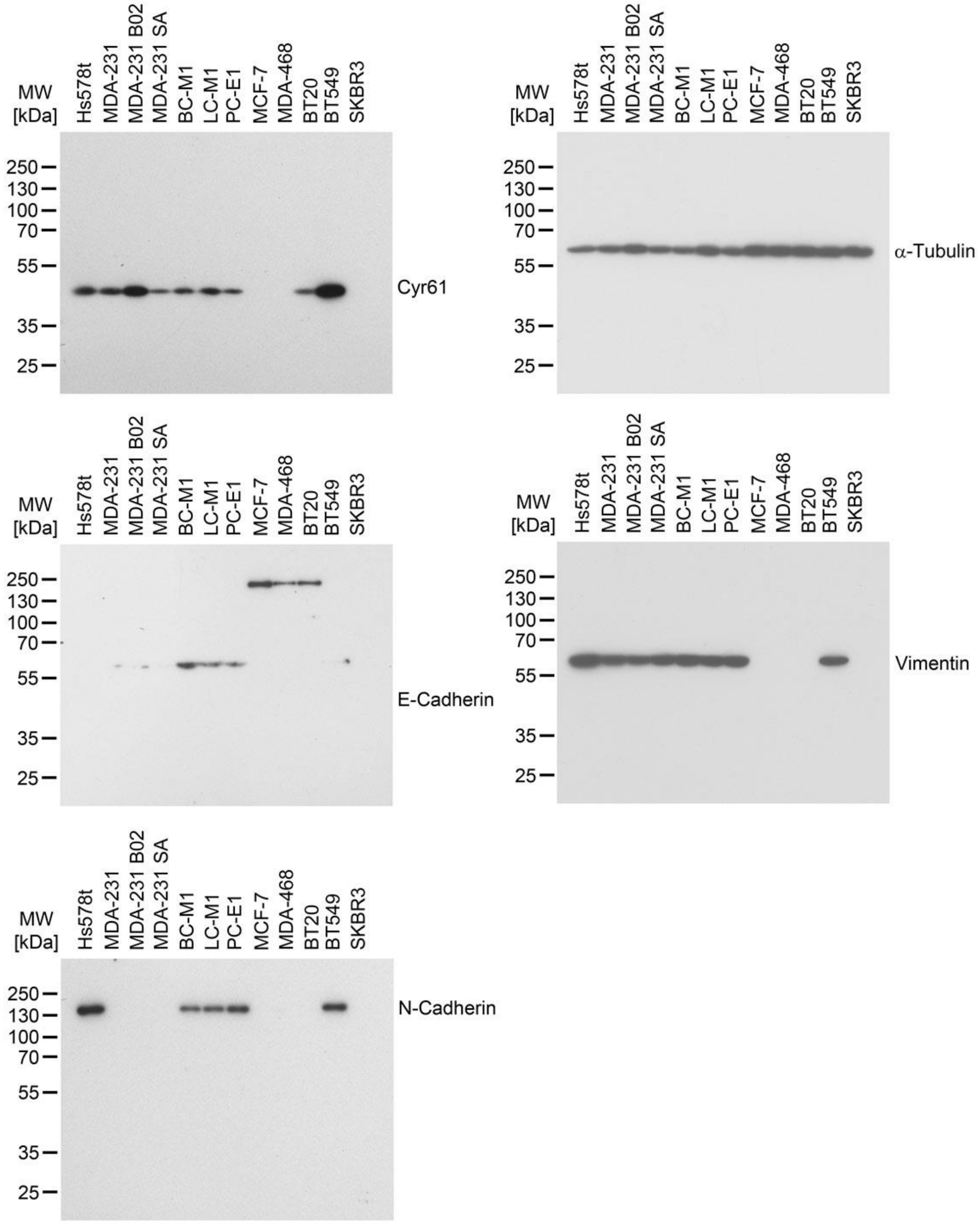

For Figure 1D:

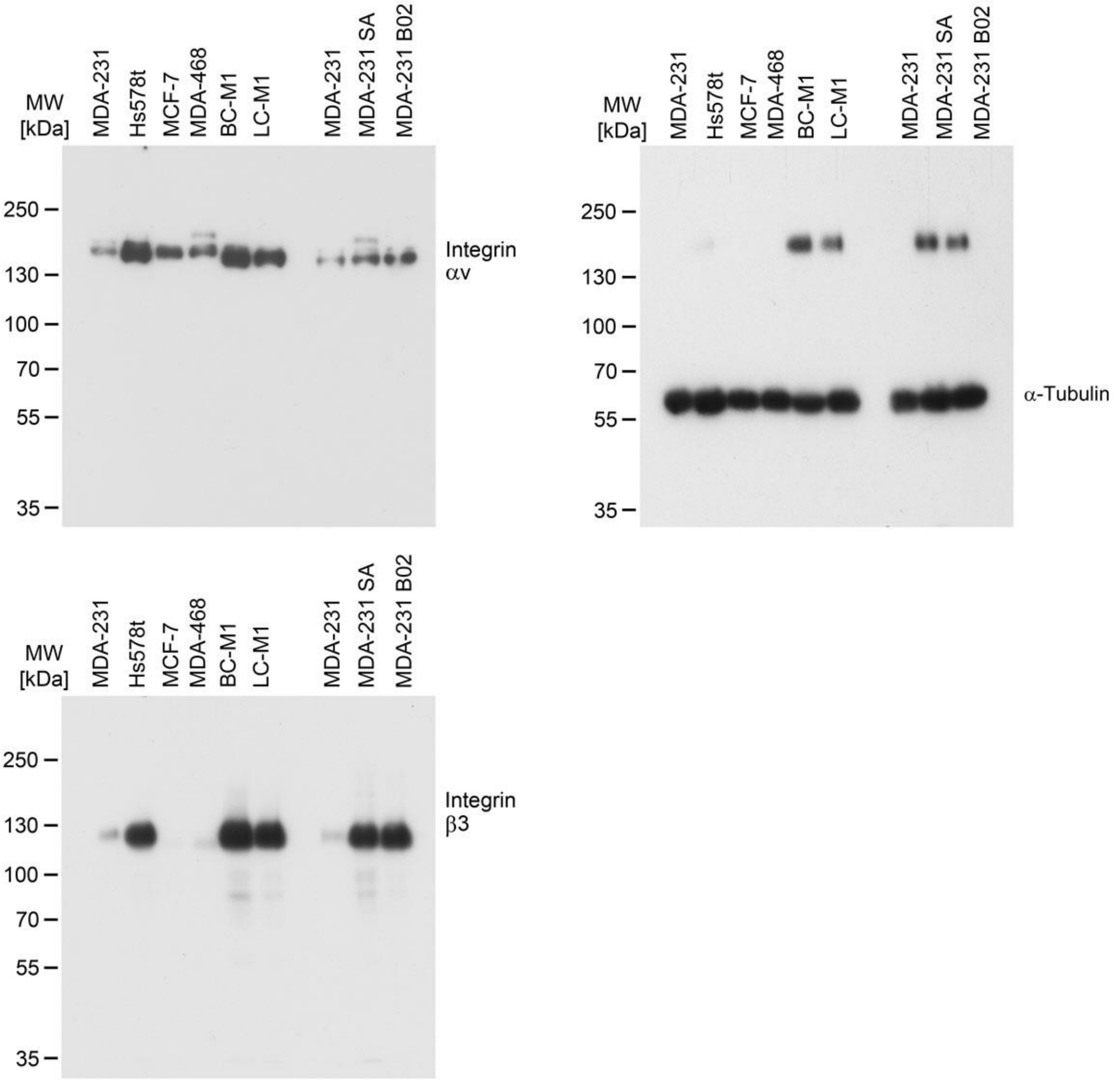

For Figure 1E:

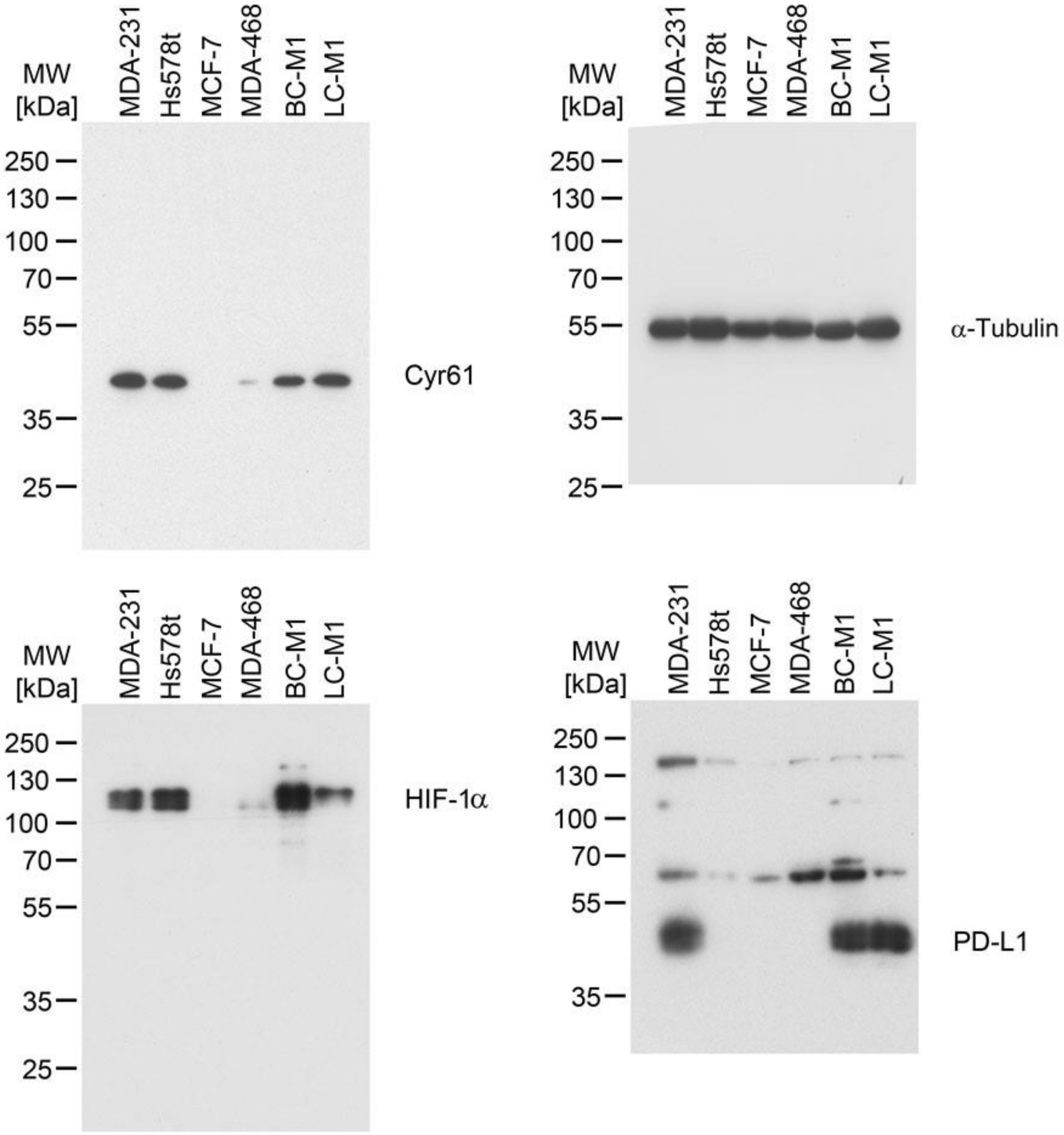

For Figure 2A:

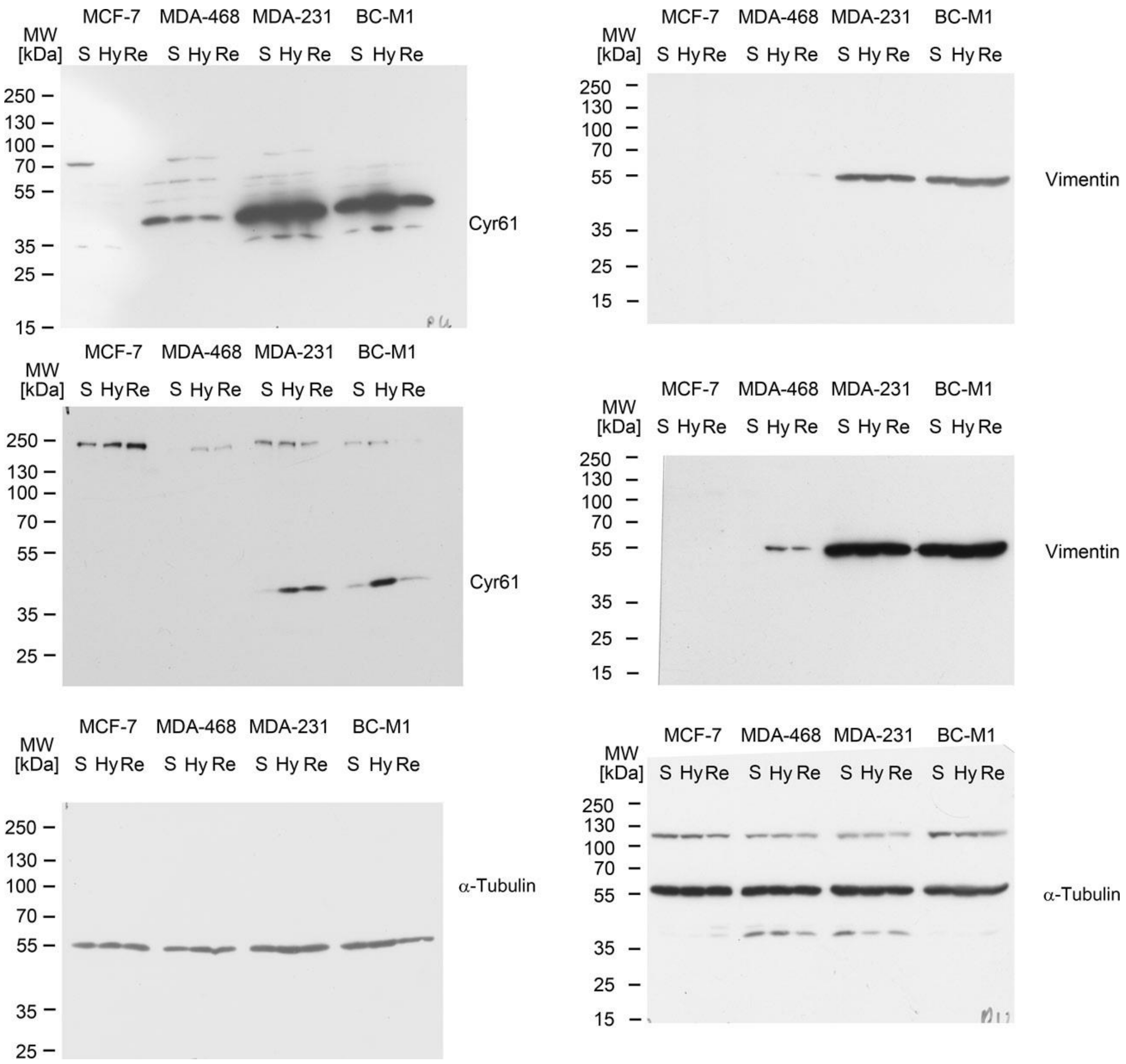

For Figure 2B:

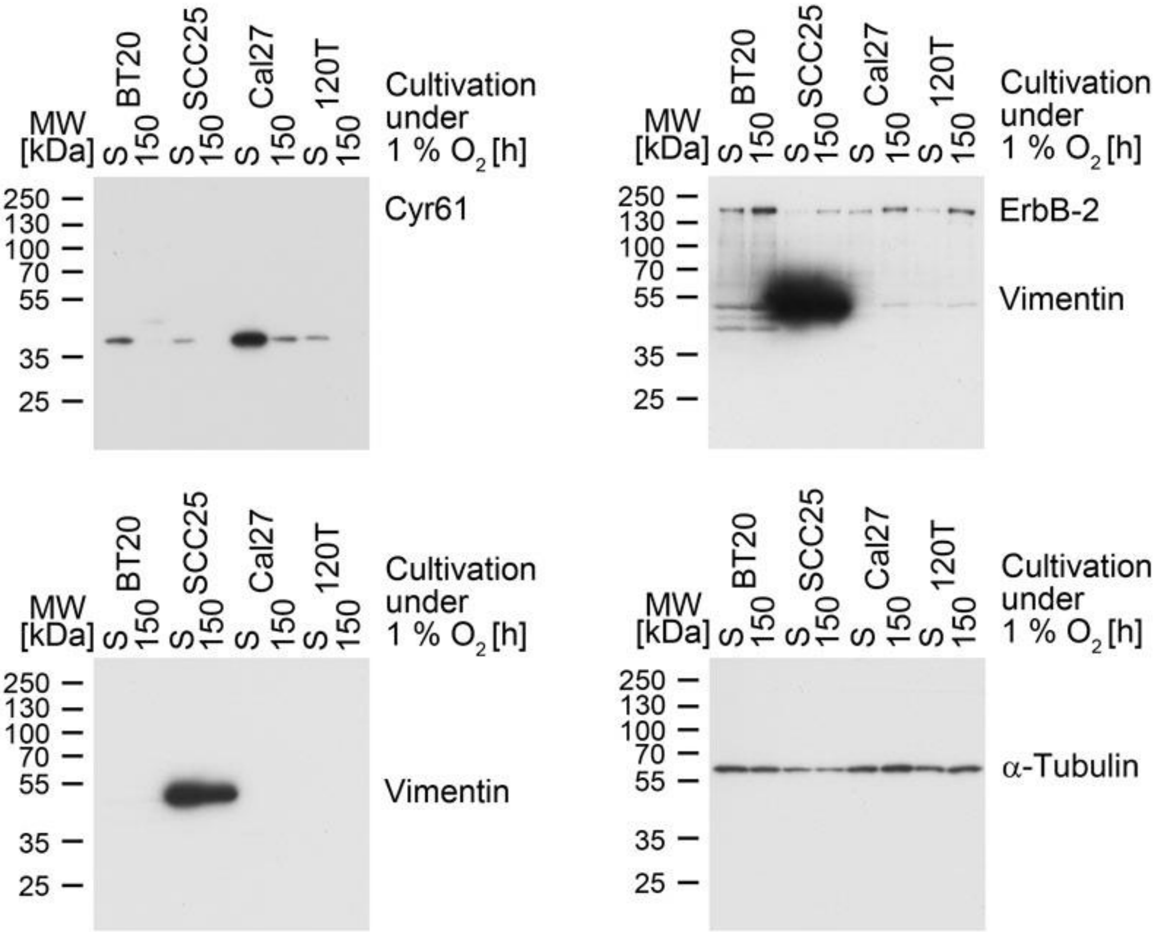

For Figure 2C:

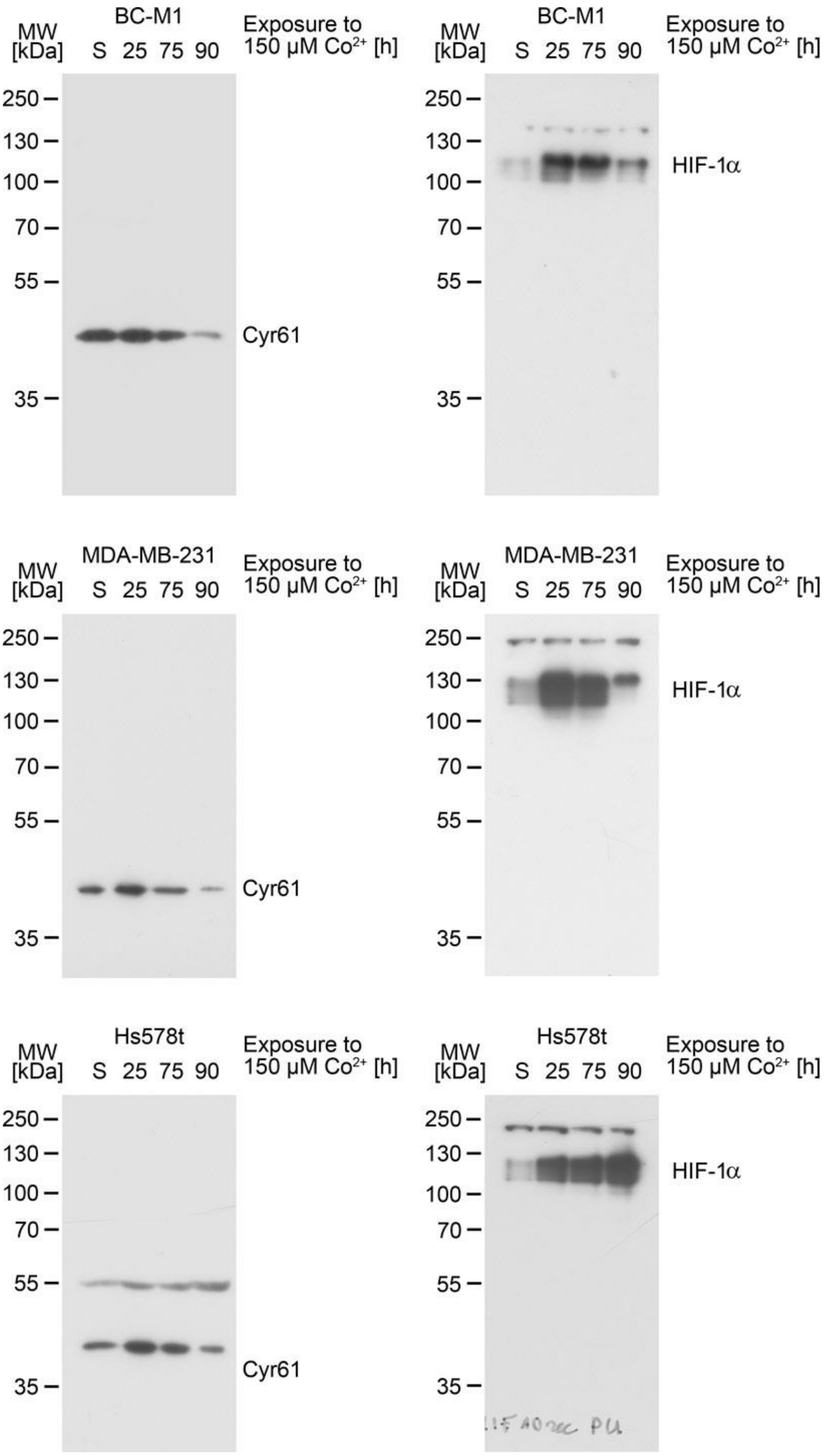

1. For Figure 2C:

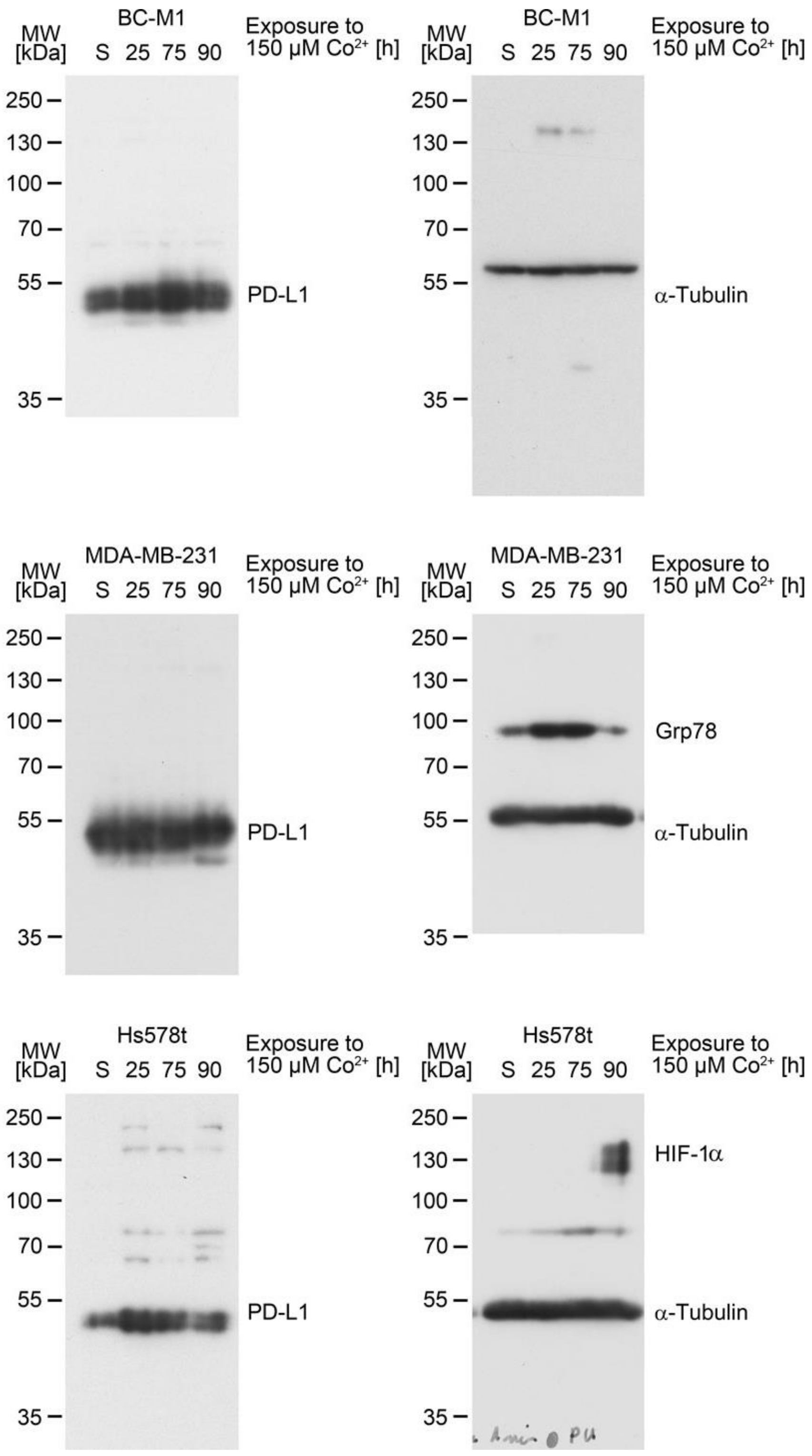

For Figure 2D:

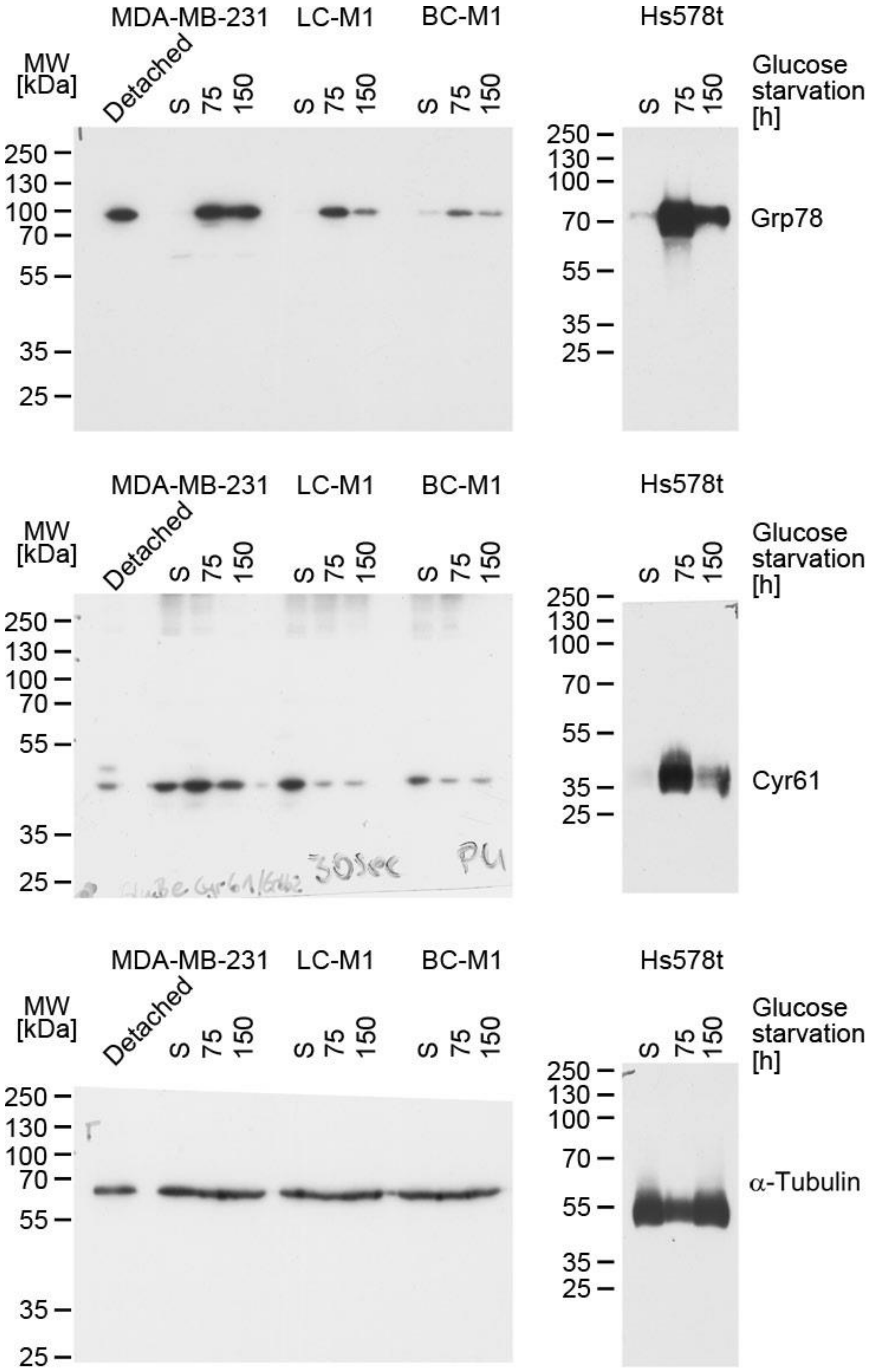

For Figure 2E:

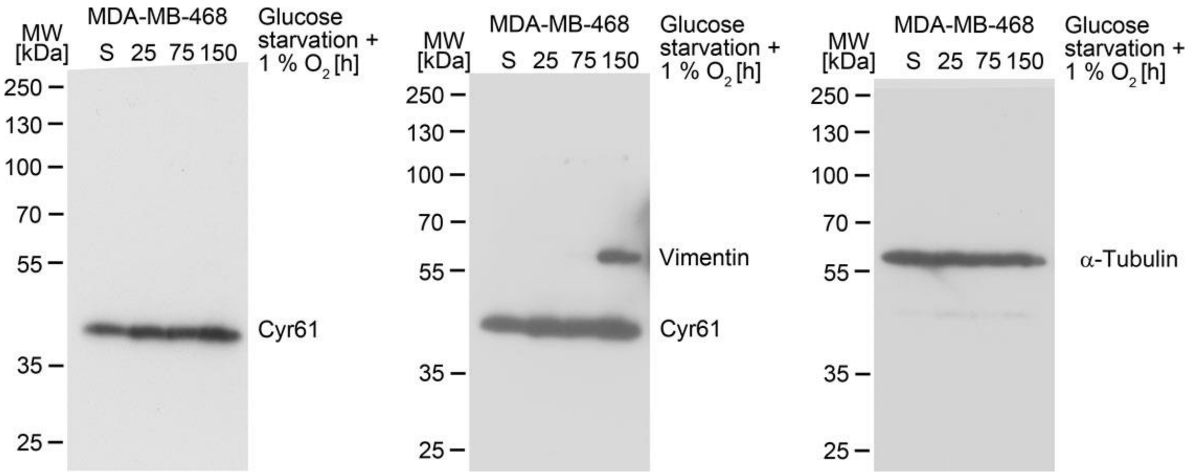

For Figure 3A:

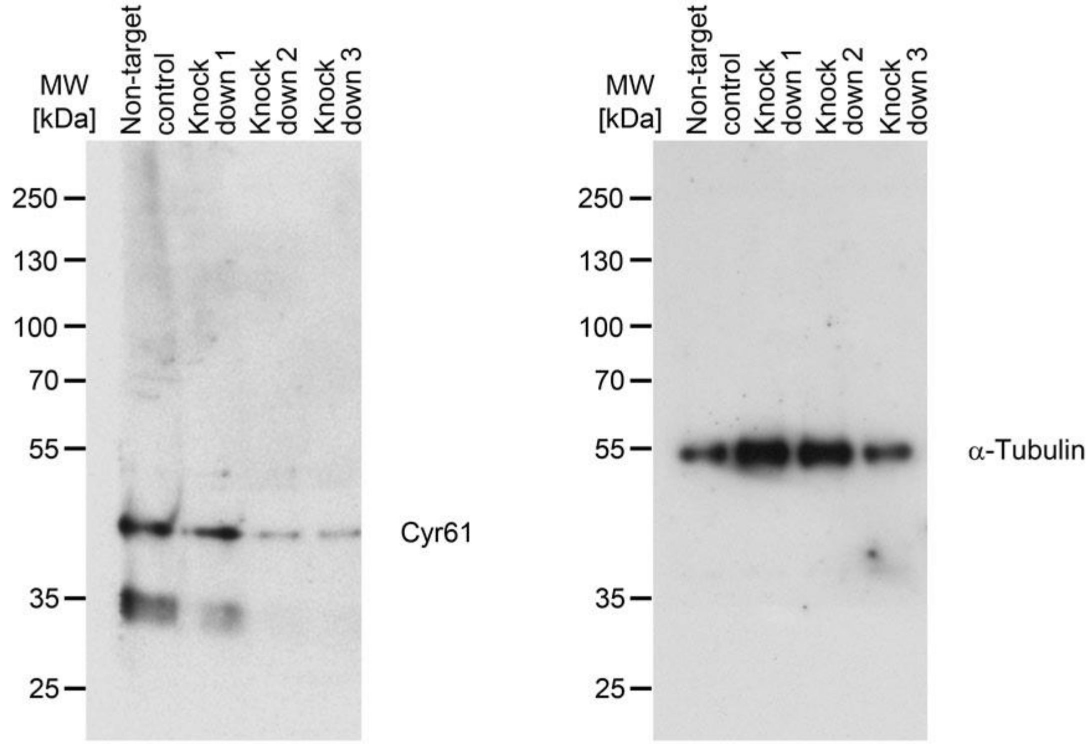

For Figure 3B:

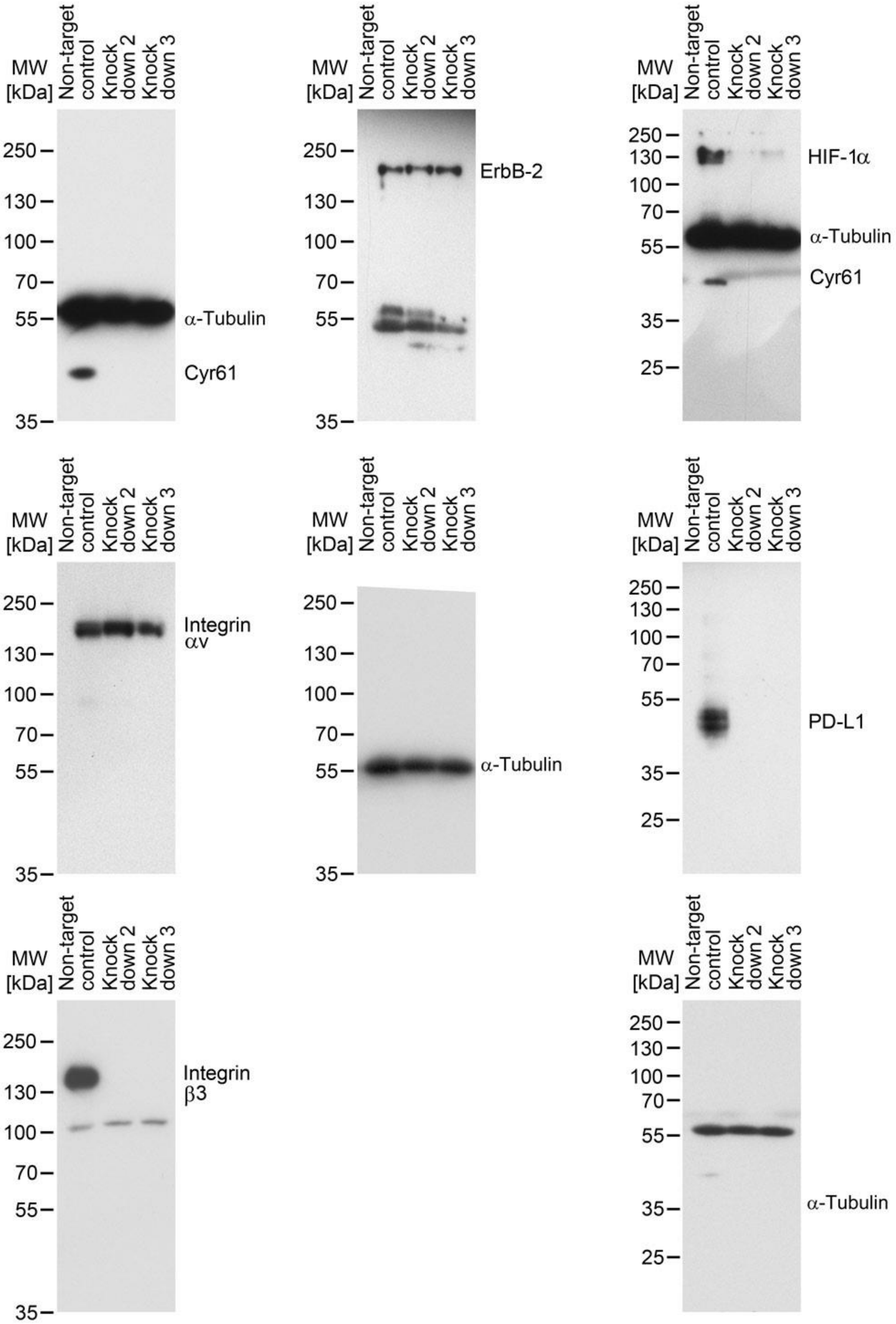

For Figure 4A:

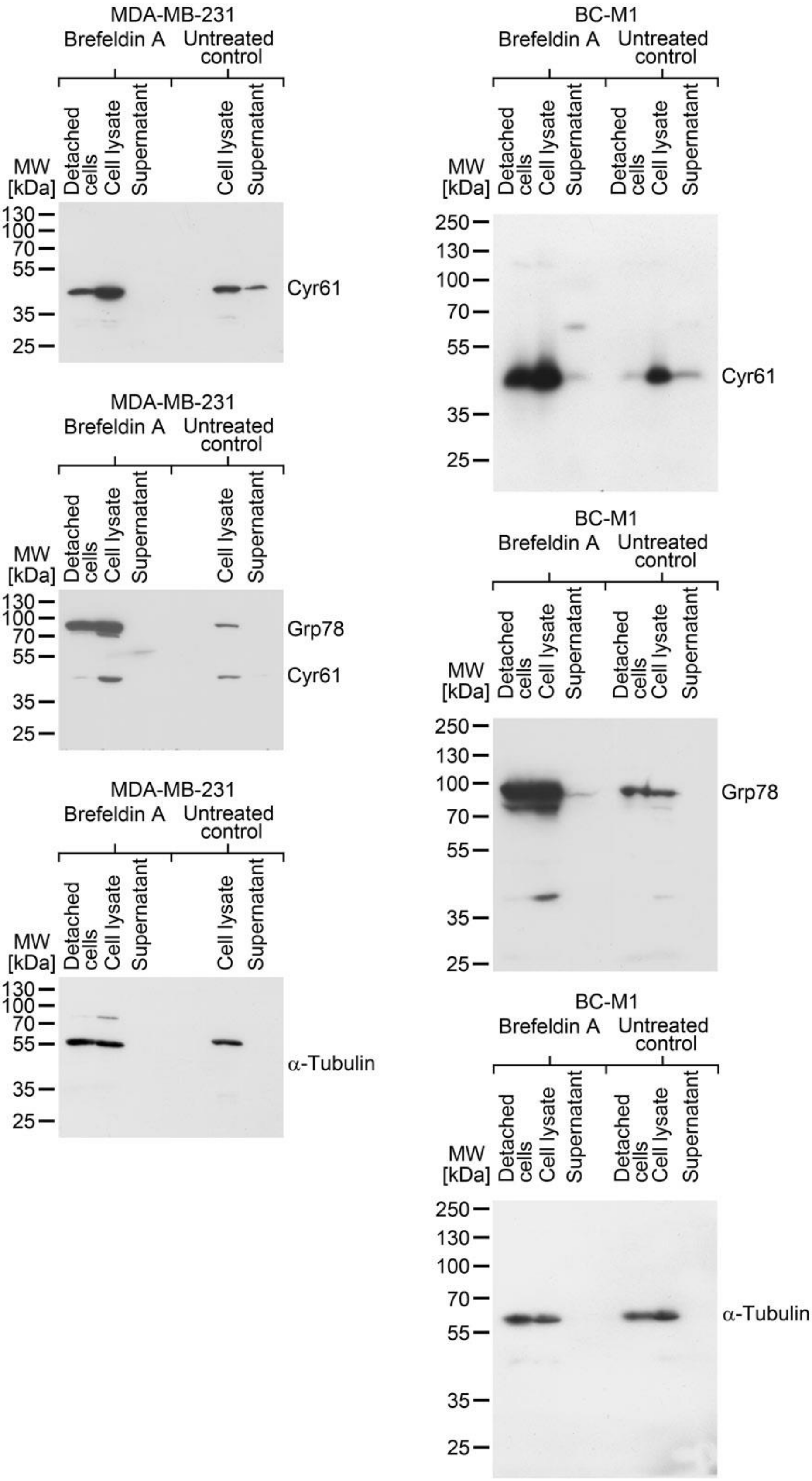

For Figure 4B:

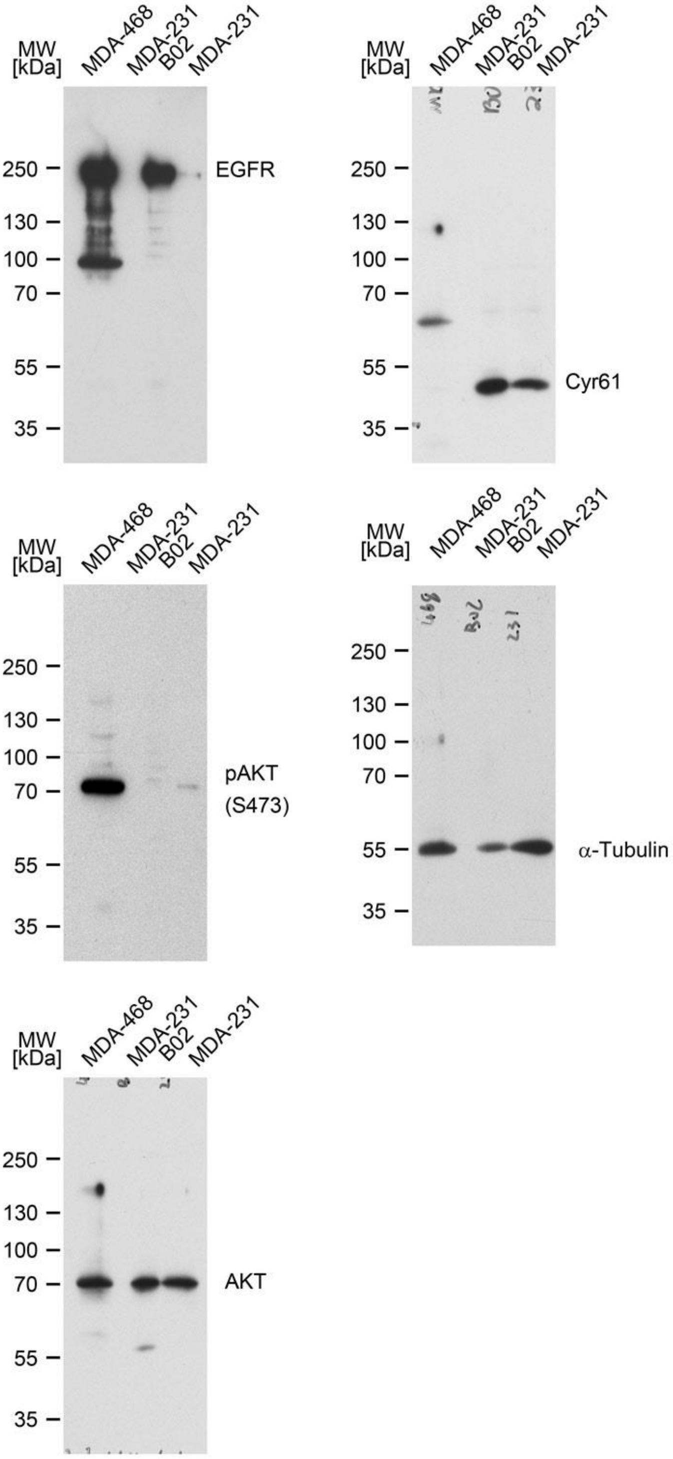

For Figure 4C:

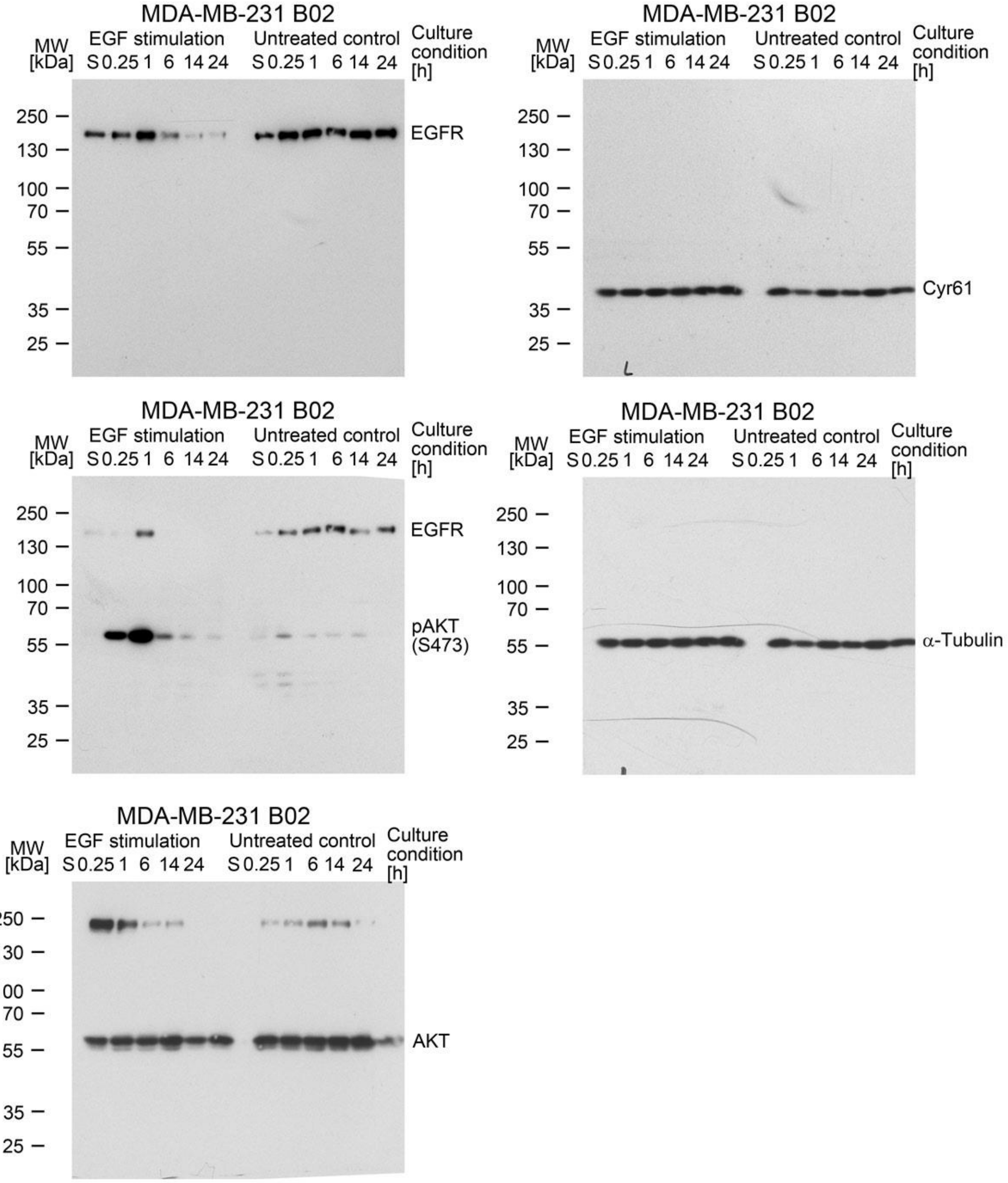

For Figure 4C:

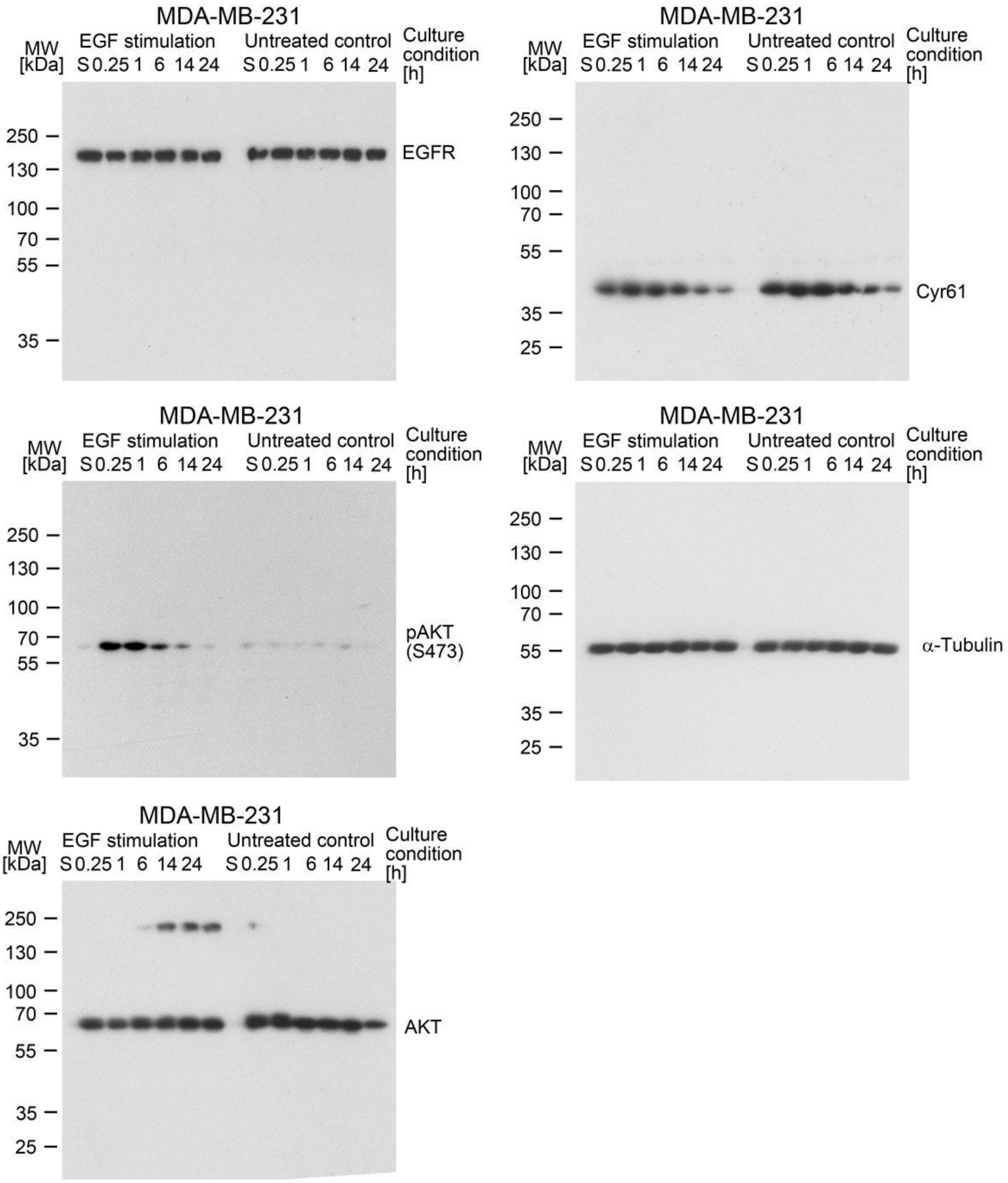

For Figure 5B:

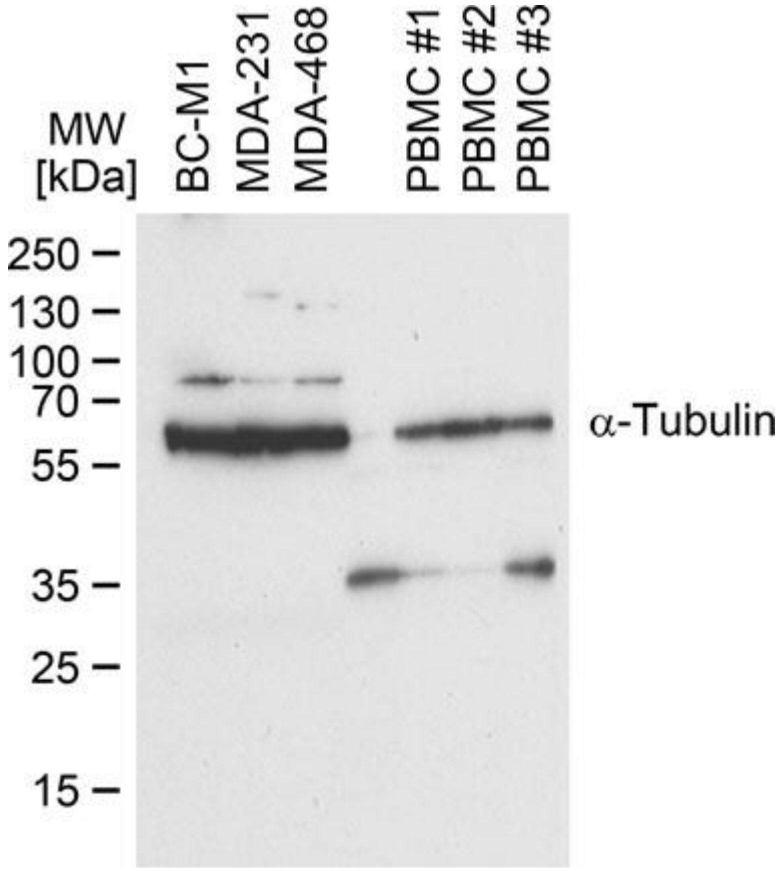

The uncropped Western Blot x-ray films for Figure 5A of the main text are in Figure S10.

© 2020 by the authors. Submitted for possible open access publication under the terms and conditions of the Creative Commons Attribution (CC BY) license (http://creativecommons.org/licenses/by/4.0/).
